# Supplementary material for: Viral modulation of type II interferon increases T cell adhesion and virus spread
Source: Nat Commun. 2024 Jun 22;15:5318. doi: 10.1038/s41467-024-49657-4 (PMC11193720; doi:10.1038/s41467-024-49657-4)

# Viral modulation of type II interferon increases T cell adhesion and virus spread

Supplementary Figure 1

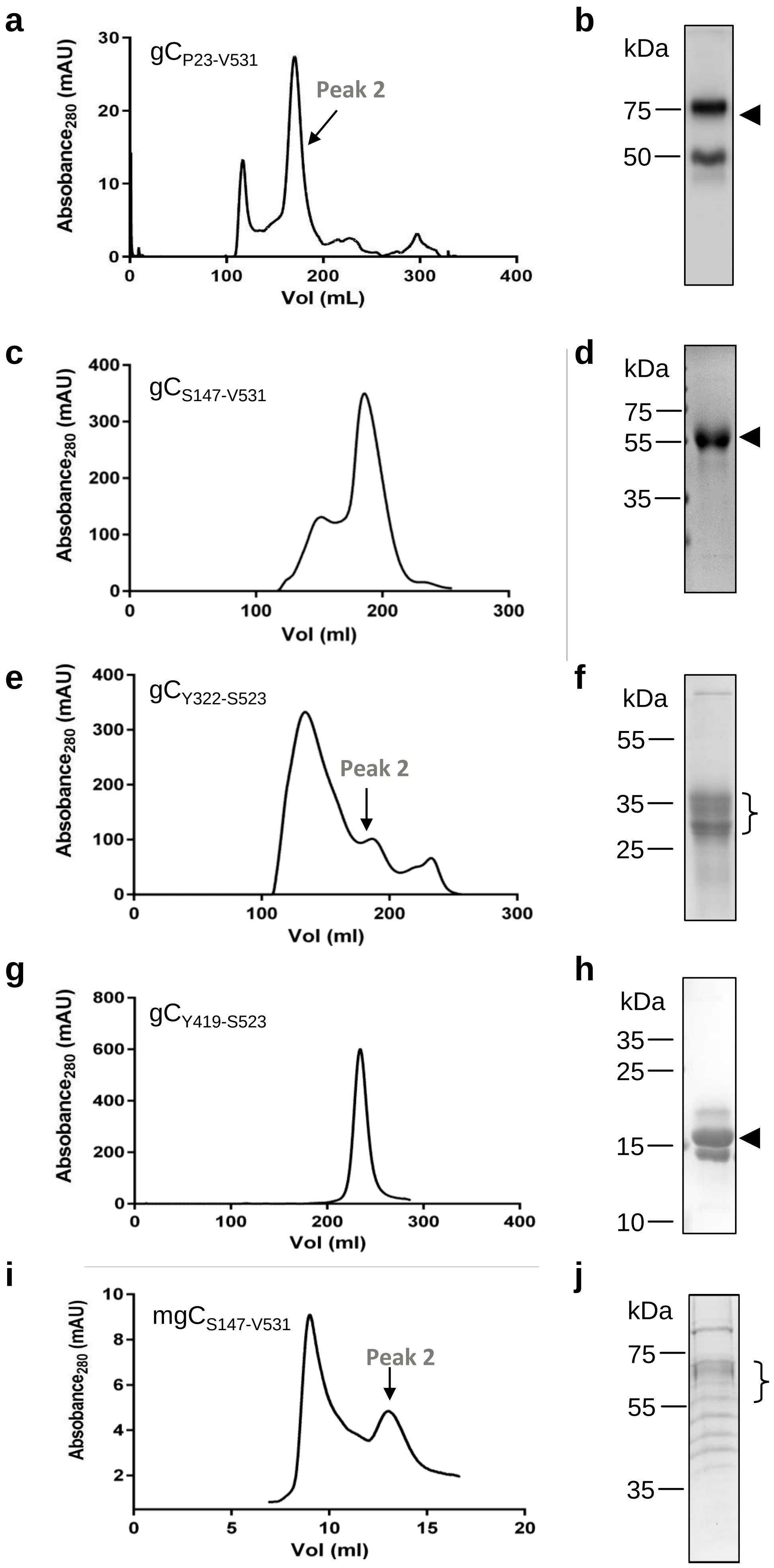

**Supplementary Figure 1. Purification of recombinant VZV gC proteins expressed in *Drosophila* S2 cells. (a, c, e, g, i)** Size-exclusion chromatography (SEC) profiles of purified recombinant $gC_{P23-V531}$ (a), $gC_{S147-V531}$ (c), $gC_{Y322-S523}$ (e) and $gC_{Y419-S523}$ (g) expressed in *Drosophila* S2 cells and $gC_{S147-V531}$ expressed in HEK293ExPi cells (i). Purification details can be found in Materials and Methods. **(b, d, f, h, j)** Images taken from SDS-PAGE loaded with purified $gC_{P23-V531}$ (b), $gC_{S147-V531}$ (d), $gC_{Y322-S523}$ (f) and $gC_{Y419-S523}$ (h) and stained for total protein with TCE (b) or Coomassie (d, f, g, j). In (b, f and j) the analyzed sample was obtained from peak 2. The molecular weight marker (in kDa) is shown on the left side. Uncropped gels are shown in Supplementary Figure 17.

# Supplementary Figure 2

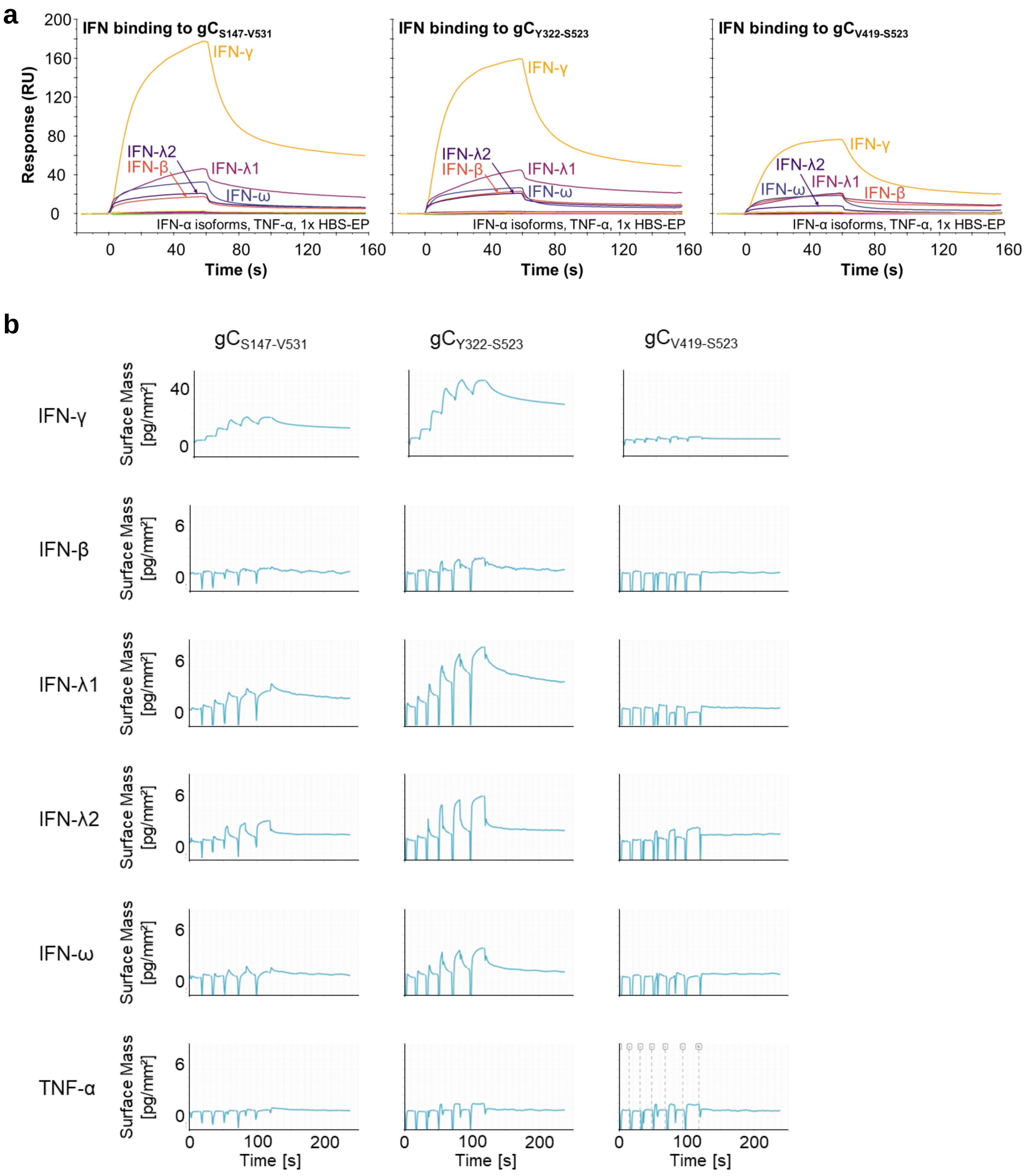

**Supplementary Figure 2. gC binds IFN-γ better than the other IFNs. (a, b)** Sensorgrams showing the results of binding screenings between VZV gC constructs and cytokines using the Biacore S200 (a) or the Creoptix WAVE (b) systems. In (a) the gC constructs were immobilized on a CM5 sensor chip (2853 RU, 1623 RU, and 668 RU, respectively) and the cytokines were injected at 100 nM with a flow rate of 30 μL/min. In (b) the different gC proteins were immobilised on DXH chips (567 pg/mm³, 584 pg/mm³, and 296 pg/mm³, respectively) and cytokines were injected at 200 nM using RAPID in the tight binder mode. Please note the different Y-axis scaling for IFN-γ compared to the other cytokines. Abbreviations: s = seconds, RU = resonance units

Supplementary Figure 3

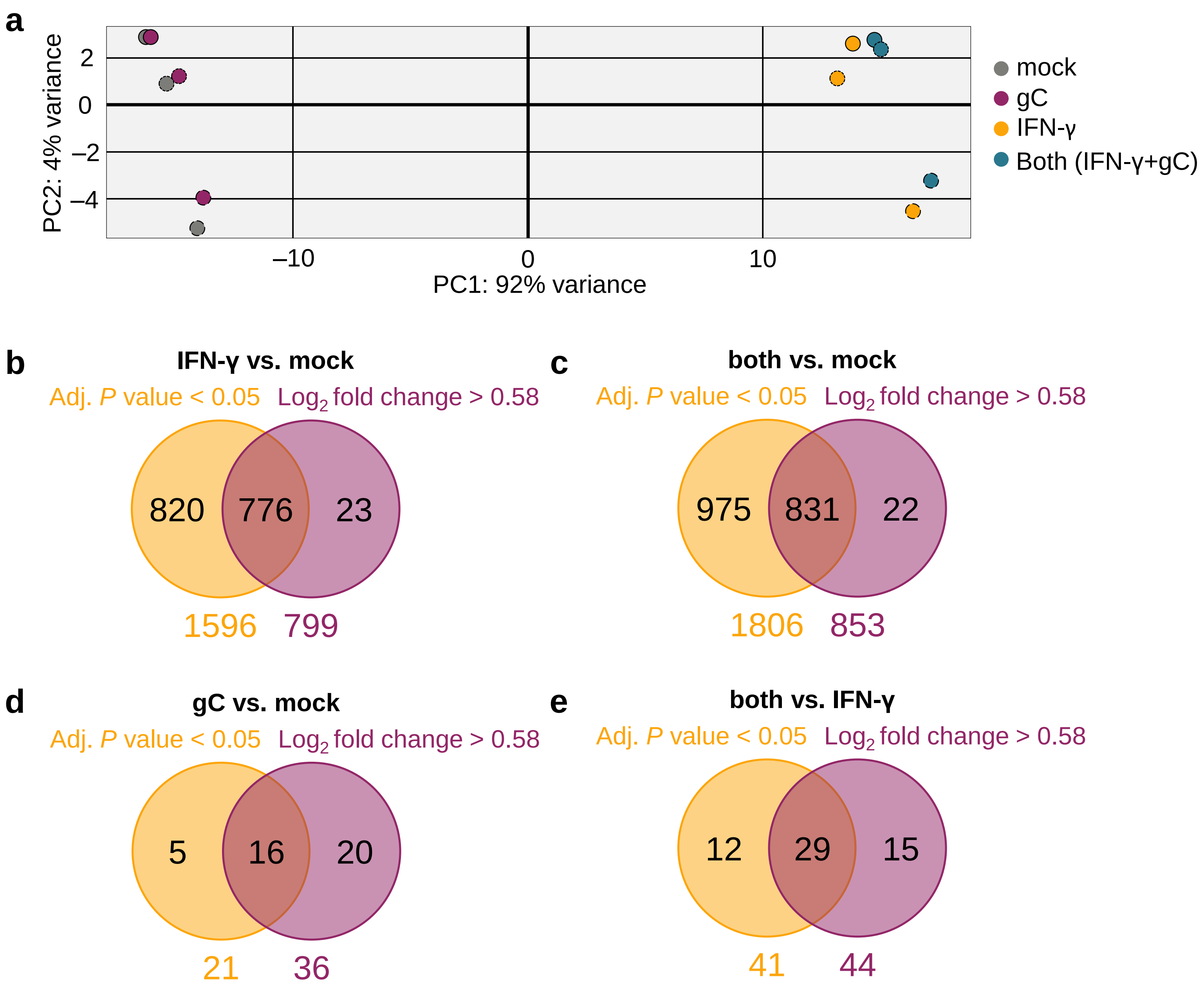

**Supplementary Figure 3. VZV gC modifies the expression of a low number of genes, including some modulated by IFN-γ.** HaCaT cells were stimulated with IFN-γ, $gC_{S147\text{-}V531}$, both or mock treated for 4 h. RNA from n=3 biological replicates was isolated and further processed for RNAseq. **(a)** Principal component analysis (PCA) of gene counts of stimulated HaCaT cells. IFN-γ had the strongest effect on variation in the samples, whereas gC only had minor effects on gene expression compared to mock. Similar boundaries of circles indicate samples obtained from the same experiment. **(b-e)** Venn diagrams showing the number of genes, whose expression level was modified at least 1.5-fold (purple) in a statistically significant manner (adjusted *P* value < 0.05 calculated with DESeq2; yellow) between the two compared conditions. Differential gene expression analysis was performed comparing the different treatment conditions. Source data are provided as Source Data 1.

Supplementary Figure 4

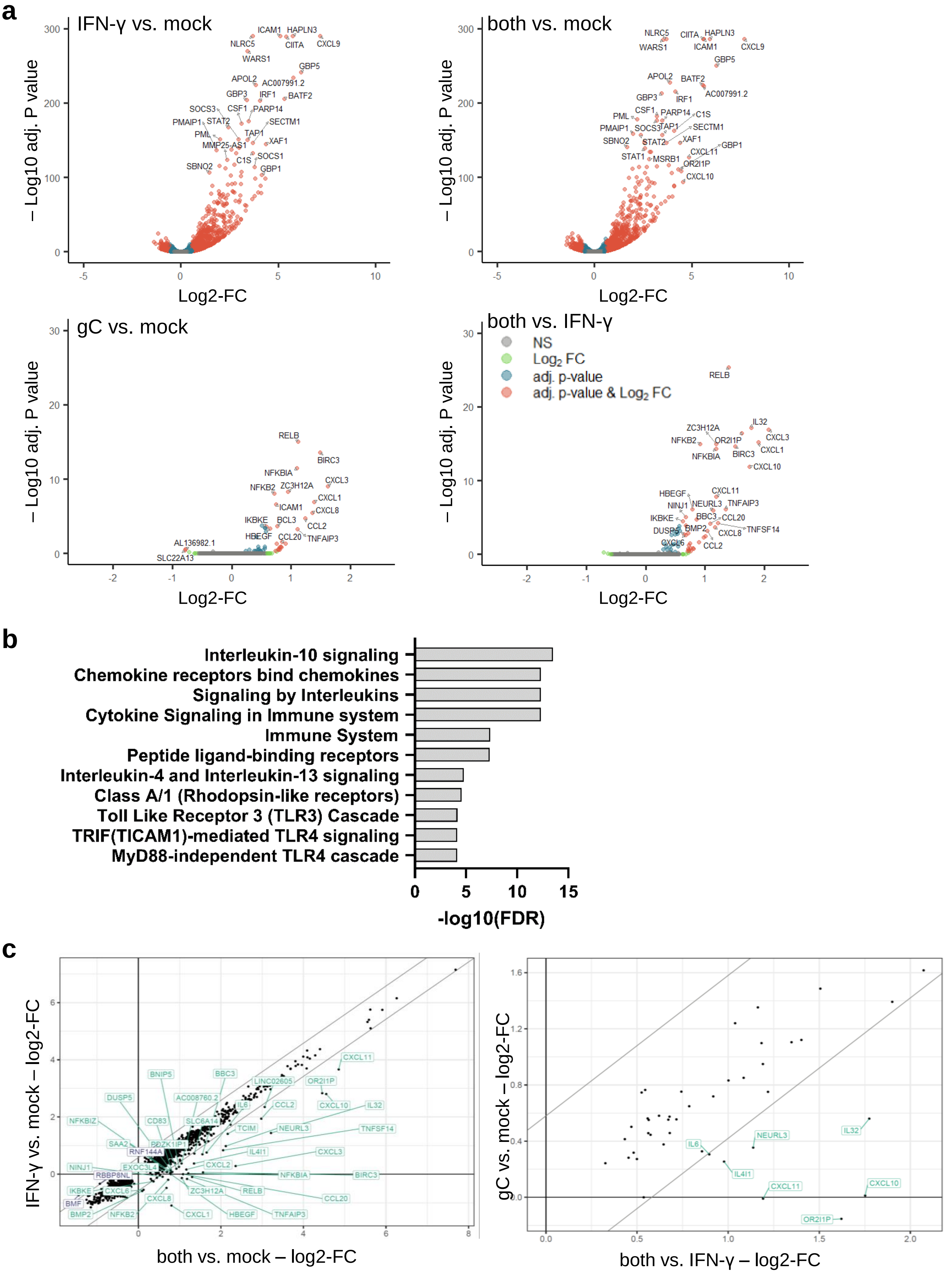

**Supplementary Figure 4. VZV gC induces ISG expression in a biased manner.** HaCaT cells were stimulated with IFN-γ, $gC_{S147-V531}$, both or mock treated for 4 h. RNA from three biological replicates (n=3) was isolated and further processed for RNAseq. Differential gene expression analysis was performed using DESeq2 comparing the different treatment conditions as indicated. **(a)** Volcano plots; x-axis represents the log2 of the fold change (FC), y-axis represents the negative decade logarithm of the adjusted (adj.) *P* value, calculated with DESeq2, for the four different comparisons. Each circle represents a gene. Boundaries were set with adj. *P* value of 0.05 and a fold change of 1.5 (equals log2-FC of 0.58). Red circles represent genes with significant change in the adj. *P* value and the fold change, blue circles depict genes with only a significant adj. *P* value, and green circles represent genes with a significant change only in the fold change. In the representation of the IFN-γ-treated versus mock control comparison, the adj. *P* values for *CXCL9*, *HAPLN3*, *NLRC5* and *ICAM1* were too low to be calculated, hence they were set to the lowest calculated adj. *P* value (*CIITA*) in this data set. In the representation of the comparison of HaCaT cells treated with IFN-γ and gC versus mock-treated cells, the adj. *P* values for *CXCL9*, *HAPLN3*, *CIITA*, *NLRC5* and *ICAM1* were too low to be calculated, hence they were set to the lowest calculated adj. *P* value (*WARS1*) in this data set. (**b**) Graph showing the pathways enriched following functional enrichment analysis of the 42 upregulated genes in the presence of gC. **(c)** Graphs showing the fold changes (FC) of significantly regulated genes from the indicated comparisons. The grey lines indicate a corridor in which genes are less than 1.5-fold changed between the two groups. Genes below the corridor are more upregulated in the comparison plotted at the x-axis (green). Genes above the corridor are stronger regulated in the comparison plotted at the y-axis (violet).

Supplementary Figure 5

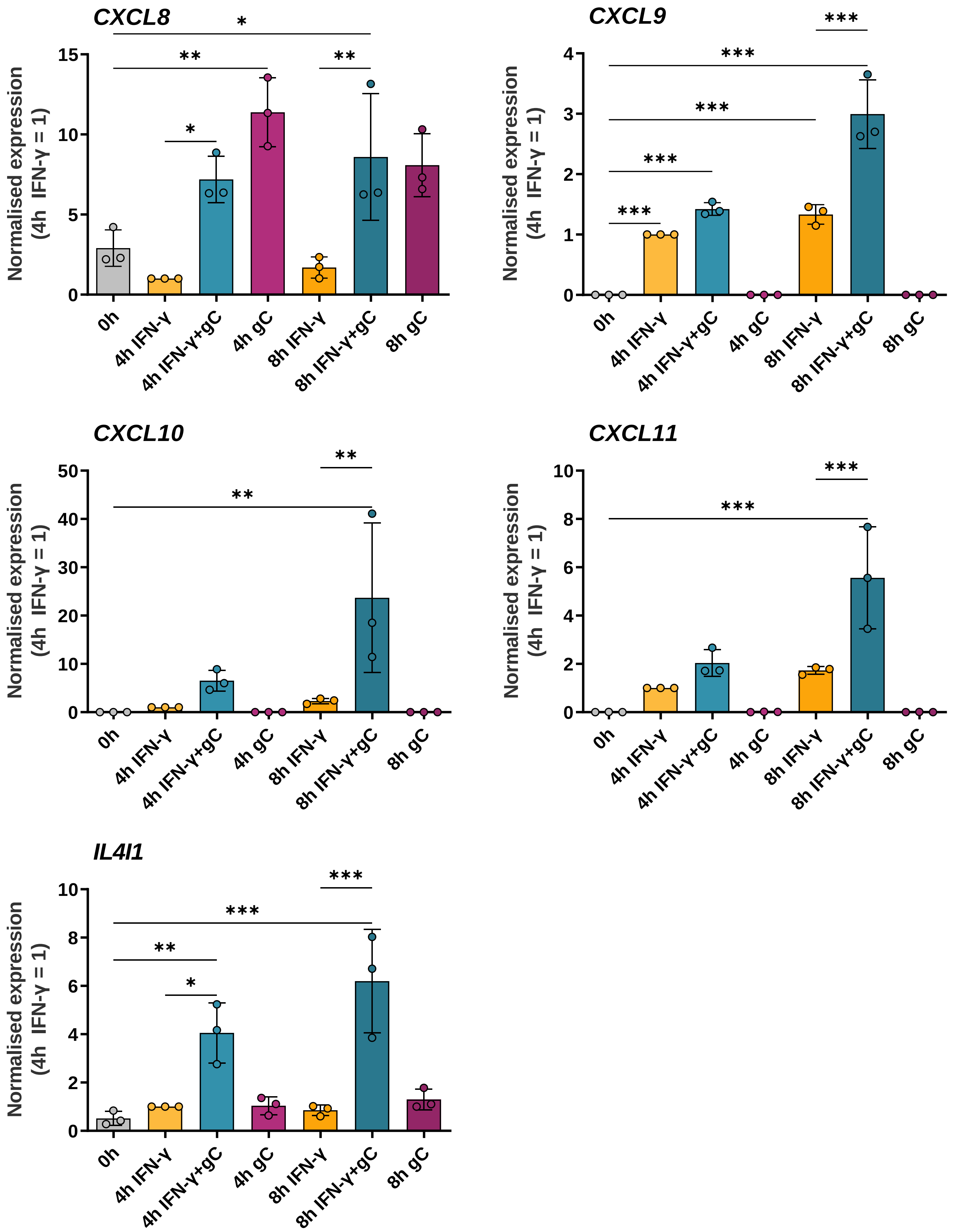

**Supplementary Figure 5. Validation of the RNASeq data by RT-qPCR.** Graphs showing expression of *CXCL8*, *CXCL9*, *CXCL10*, *CXCL11* and *IL4I1* relative to *actin* in HaCaT cells stimulated with 5 ng/mL IFN-γ, 300 nM $gC_{S147\text{-}V531}$ or both for the indicated time points. The values were normalized to those obtained from cells stimulated for 4 h with IFN-γ. Each filled circle corresponds to one independent assay (n=3 biological replicates). Error bars represent mean ± SD from three biological replicates. One-way ANOVA was performed, followed by Šídák's multiple comparisons (comparing each sample to the mock and between IFN-γ stimulated cells vs. co-stimulated cells for each time point). Non-significant comparisons are not indicated. * = $P$ <0.033; ** = $P$ <0.002; *** = $P$ <0.001.

Supplementary Figure 6

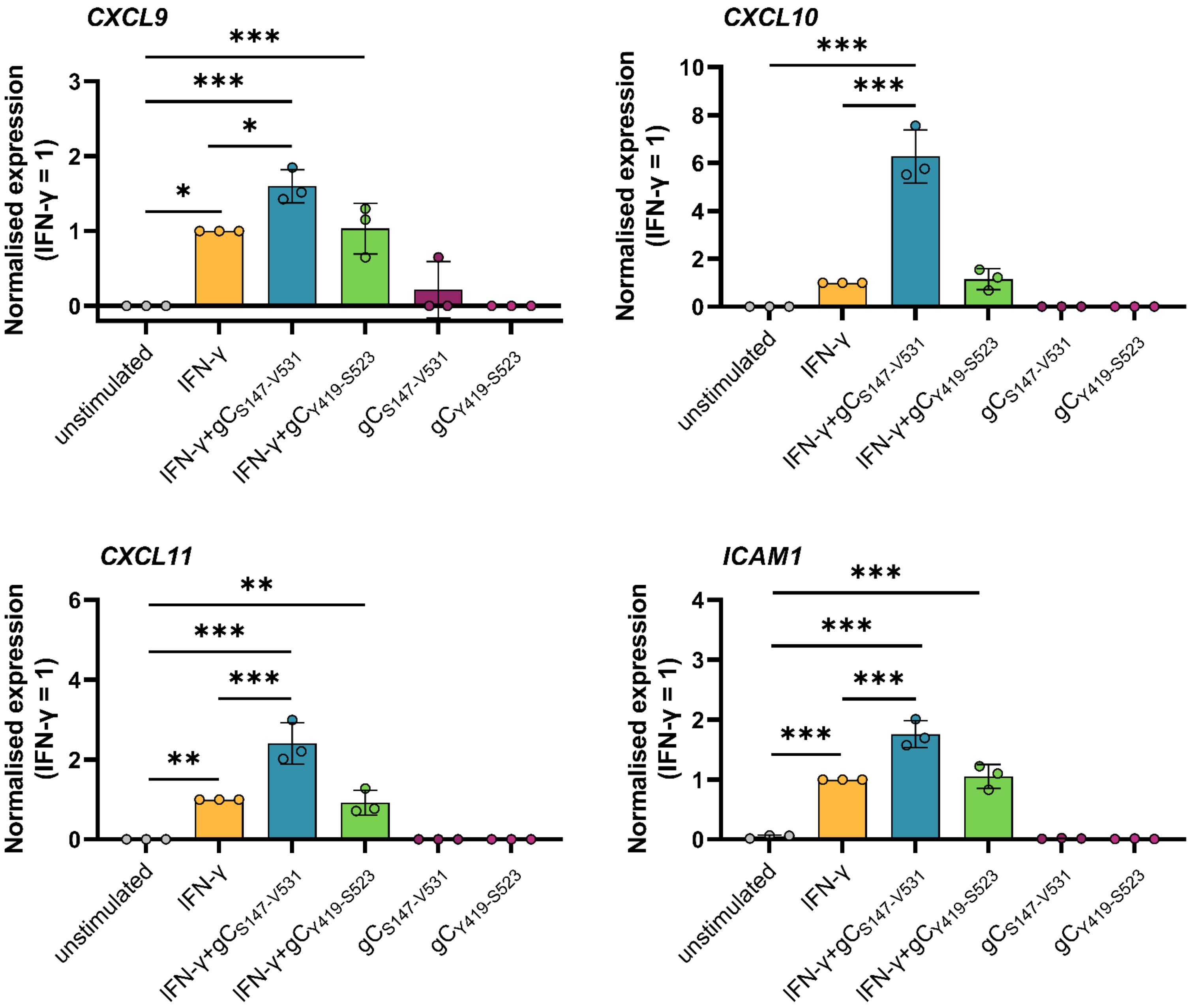

**Supplementary Figure 6. $gC_{Y419\text{-}S523}$ does not induce the expression of ISGs as efficiently as $gC_{S147\text{-}V531}$**. Graphs showing expression of *CXCL9*, *CXCL10*, *CXCL11* and *ICAM1* relative to *actin* in HaCaT cells stimulated with 5 ng/mL IFN-γ and/or 300 nM $gC_{S147\text{-}V531}$ or $gC_{Y419\text{-}S523}$ for 8 hours. The values were normalized to those obtained from cells stimulated with IFN-γ only. Each filled circle corresponds to one independent assay (n=3 biological replicates). Error bars represent mean ± SD from three biological replicates. One-way ANOVA was performed, followed by Šídák's multiple comparisons (comparing each sample to the unstimulated one and between IFN-γ stimulated cells vs. co-stimulated cells). Non-significant comparisons are not indicated. * = $P$ <0.033; ** = $P$ <0.002; *** = $P$ <0.001.

# Supplementary Figure 7

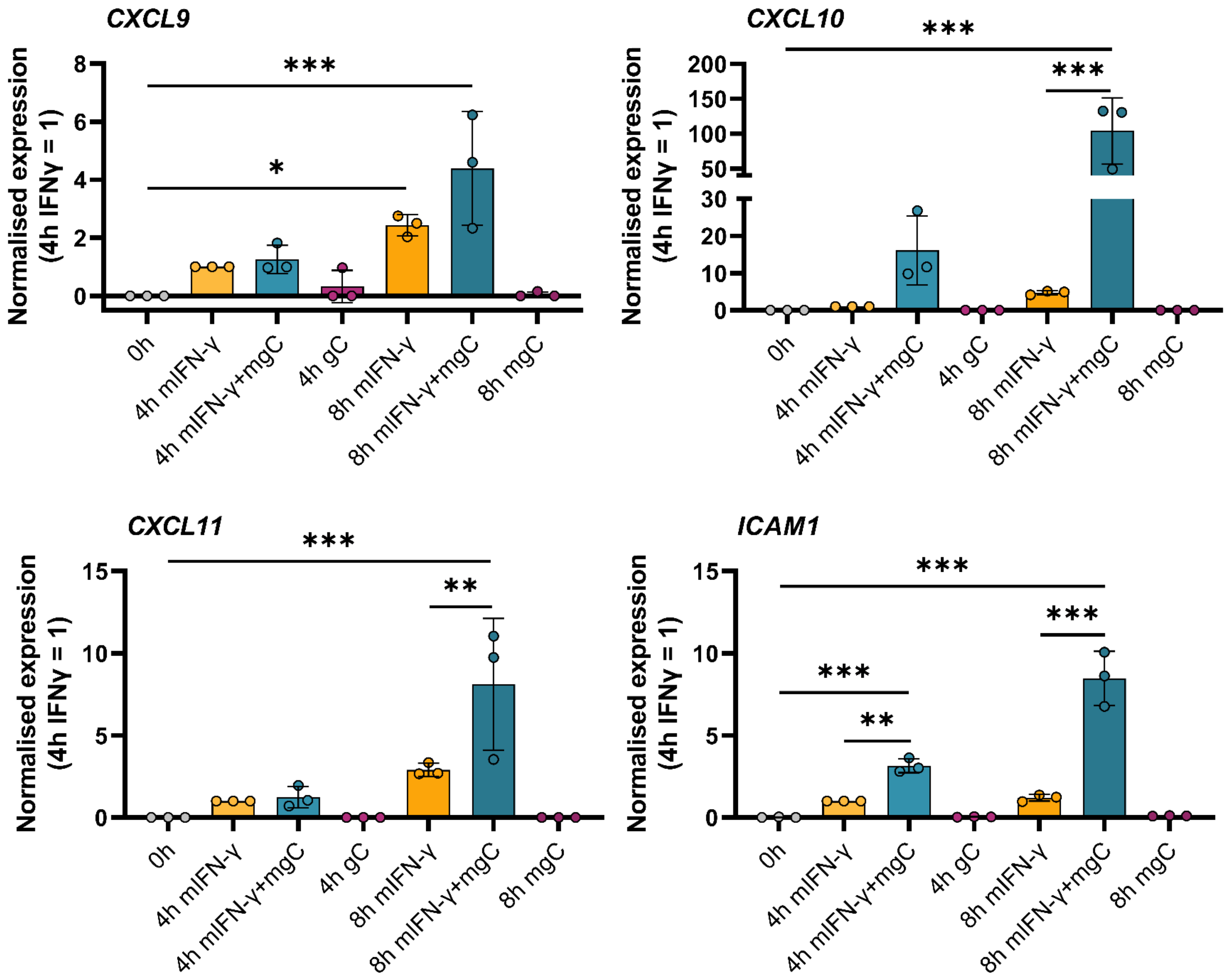

**Supplementary Figure 7. gC$_{S147\text{-}V531}$ increases IFN-γ-mediated ISG expression when both proteins are purified from mammalian cells.** Graphs showing expression of *CXCL9*, *CXCL10*, *CXCL11* and *ICAM1* relative to *actin* in HaCaT cells stimulated with mammalian (m) expressed mIFN-γ (5 ng/mL), mgC$_{S147\text{-}V531}$ (300 nM) or both for the indicated time points. The values were normalized to those obtained from cells stimulated for 4 h with mIFN-γ. Each filled circle corresponds to one independent assay (n=3 biological replicates). Error bars represent mean ± SD from three biological replicates. One-way ANOVA was performed, followed by Šídák's multiple comparisons (comparing each sample to the mock and between IFN-γ stimulated cells vs. co-stimulated cells for each time point). Non-significant comparisons are not indicated. * = $P$ <0.033; ** = $P$ <0.002; *** = $P$ <0.001.

Supplementary Figure 8

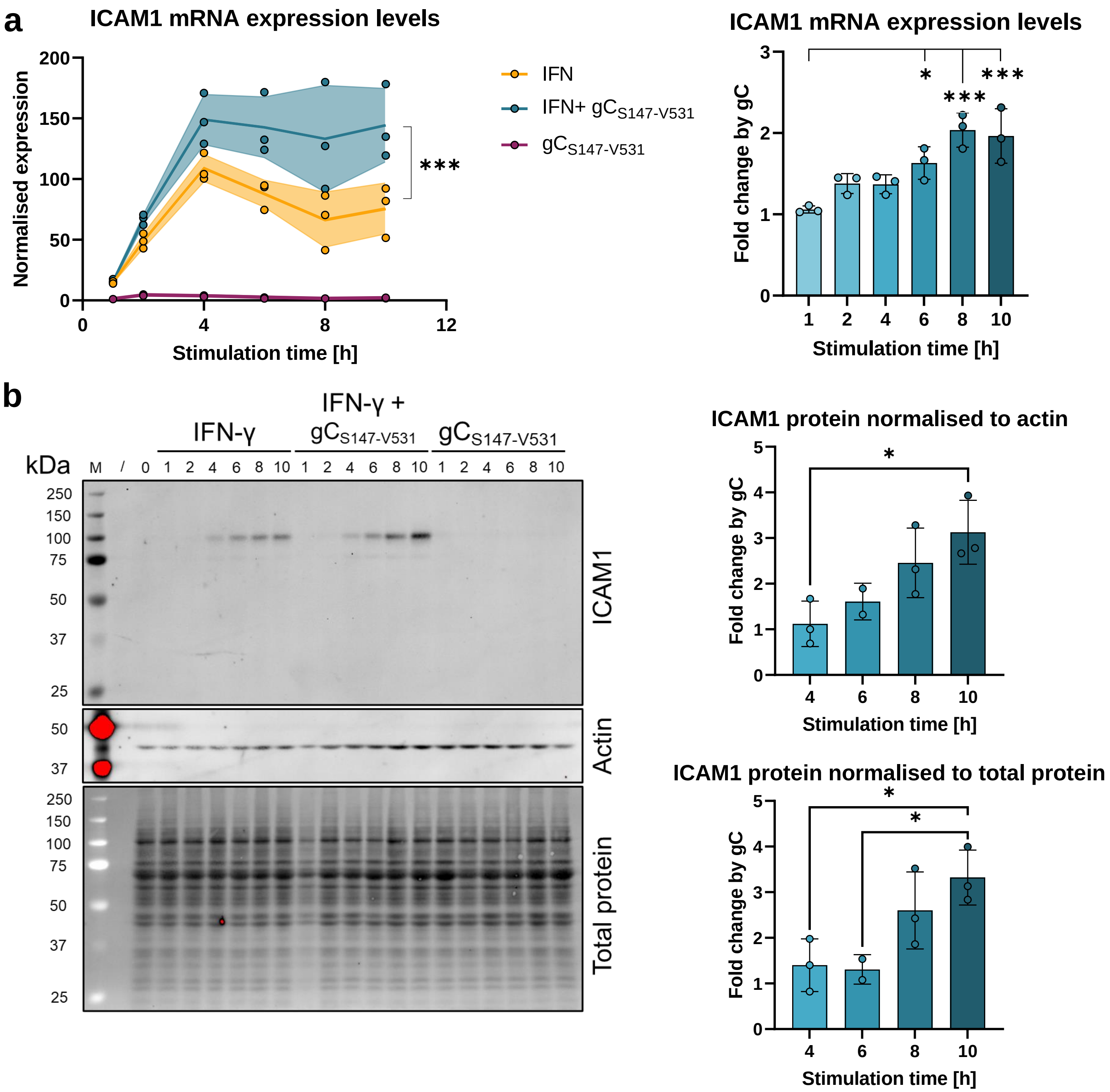

**Supplementary Figure 8. HaCaT cells co-stimulated with VZV gC and IFN-γ show increased mRNA expression and total ICAM1 protein levels. (a)** HaCaT cells were stimulated with 5 ng/mL IFN-γ and/or 300 nM $gC_{S147\text{-}V531}$ for the indicated time points, RNA was isolated and analyzed for *ICAM1* expression by RT-qPCR. Using the ΔΔCt method, *ICAM1* level was normalized to unstimulated cells and to *actin* as housekeeping gene. Shown are the individual values from three independent assays (n=3 biological replicates) (filled circles), the resulting mean is shown as solid line with standard deviation (SD) as colored transparent background. The area under the curve (AUC) was calculated, then one-way ANOVA was performed on the AUC values, followed by Dunnett's multiple comparisons. The fold change induced by addition of $gC_{S147\text{-}V531}$ to IFN-γ was calculated for each timepoint and plotted in a bar chart (right panel). Filled circles represent the individual values from each independent experiment, bars represent the mean ± SD. One-way ANOVA, followed by Dunnett's multiple comparisons was performed (comparing to the 1 h stimulation time). **(b)** Immunoblots detecting ICAM1 (top panel) and actin (middle panel) and blot showing total protein signal visualized using TCE (bottom panel) in same samples employed in (a). One representative experiment out of three biological experiments is shown. Quantification of the western blot band intensities from three independent experiments is shown in the right panels. Top panel shows the normalization of ICAM1 signal to actin, bottom panel shows ICAM1 signal normalized to total protein. Shown are mean ± SD. One-way ANOVA, followed by Tukey's multiple comparisons was performed (comparing all samples to each other). ns = not significant; * = $P$ <0.033; ** = $P$ <0.002; *** = $P$ <0.001. Abbreviations: kDa = kilo Dalton; M = marker; h = hours

# Supplementary Figure 9

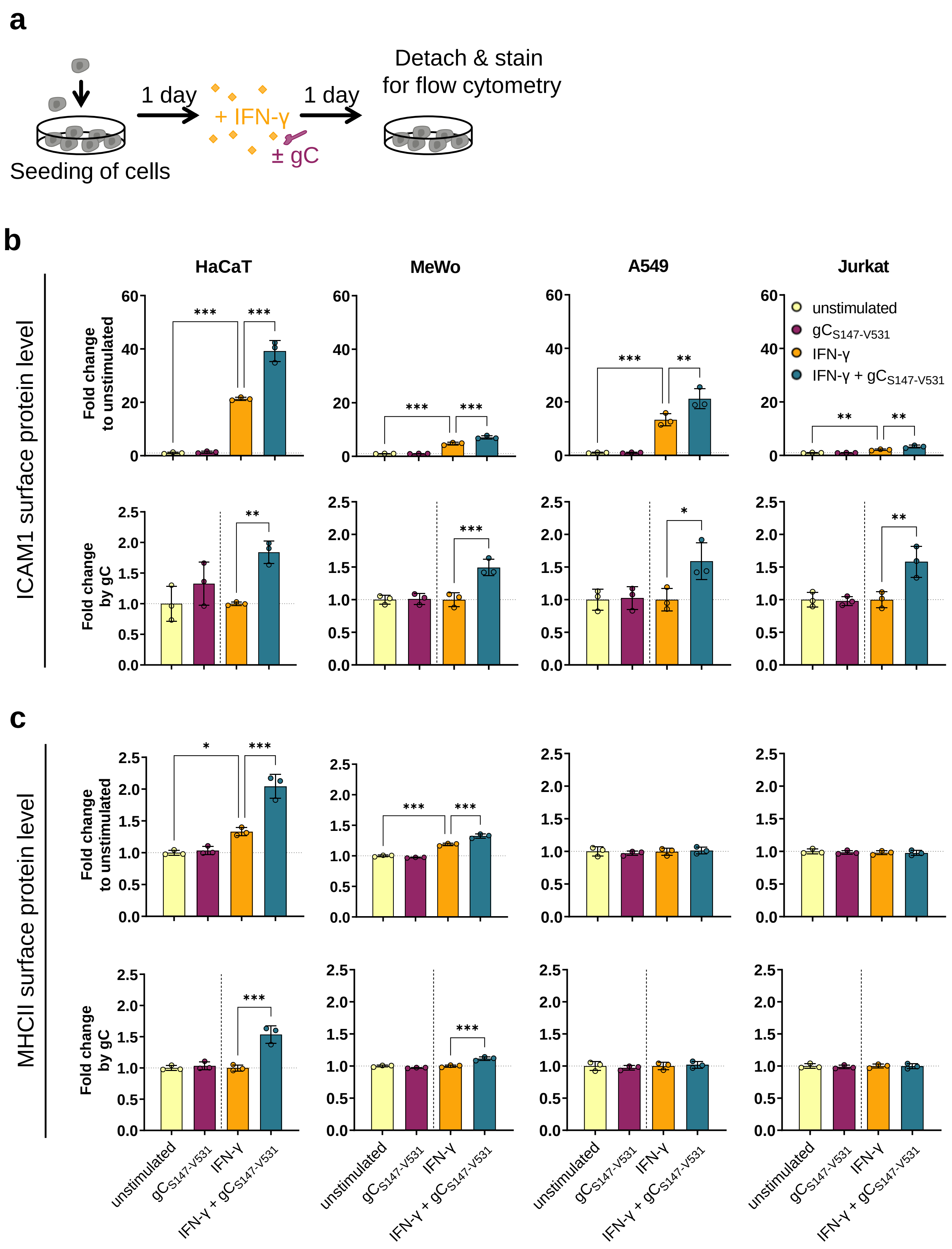

**Supplementary Figure 9. Cell lines co-stimulated with VZV gC and IFN-γ have increased surface levels of ICAM1. (a)** Schematic representation of the assay. Cells were seeded one day prior to stimulation with 5 ng/mL IFN-γ, 300 nM VZV $gC_{S147\text{-}V531}$ or both. 24 h after stimulation the cells were detached, stained, and analyzed by flow cytometry to detect ICAM1 (b) of MHCII (c) at the plasma membrane. **(b, c)**. Bar charts showing the fold change of ICAM1 (b) or MHCII (c) surface protein levels compared to unstimulated cells (top row) or induced by $gC_{S147\text{-}V531}$ compared to either mock or IFN-γ baseline (bottom row). The median fluorescence intensities were determined after gating on single and alive cells. Bars show the mean ± SD, filled circles represent values from three independent experiments (n=3 biological replicates). One-way ANOVA, followed by Šídák's multiple comparisons was performed (comparison IFN-γ to unstimulated and/or condition with gC to baseline without gC). Non-significant comparisons are not indicated. * = $P < 0.033$; ** = $P < 0.002$; *** = $P < 0.001$.

# Supplementary Figure 10

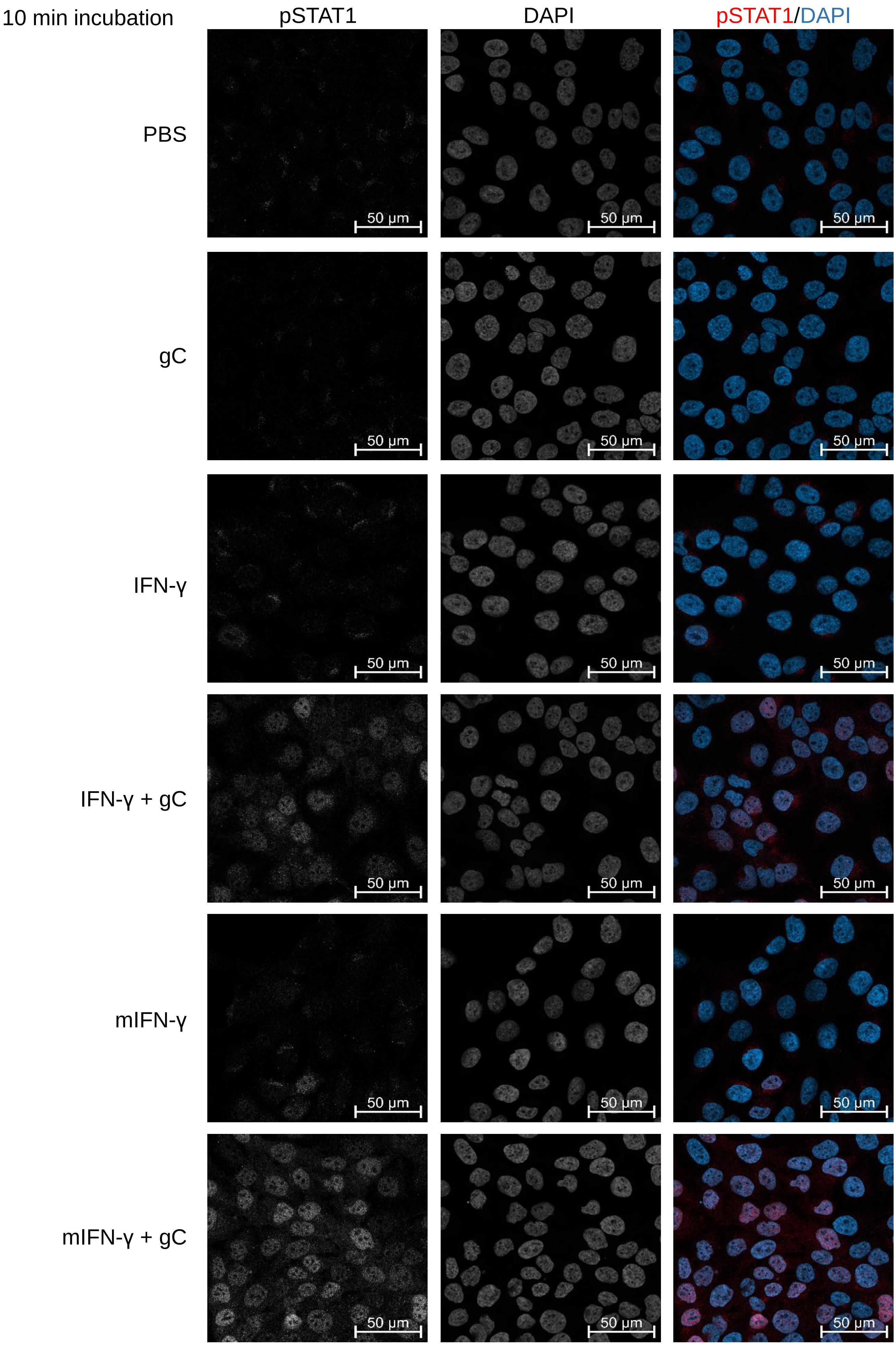

**Supplementary Figure 10. gC$_{S147\text{-}V531}$ increases IFN-γ-mediated nuclear translocation of p-STAT1 at 10 minutes post-incubation.** Immunofluorescence images showing p-STAT1 in the cytoplasm and nucleus of HaCaT cells incubated during 10 minutes at 37 °C with PBS, gC$_{S147\text{-}V531}$, IFN-γ expressed in bacteria or in mammalian cells (mIFN-γ) or both gC$_{S147\text{-}V531}$ and IFN γ. p-STAT1 was detected with a rabbit monoclonal anti-p-STAT1 Y701 antibody and nuclei were labelled with DAPI. The first two columns show the individual channels in grey while the third column shows both p-STAT1 (red) and DAPI (blue). n=2 biological replicates, except for mIFN-γ, which was performed only once. Scale bar 50 μm.

# Supplementary Figure 11

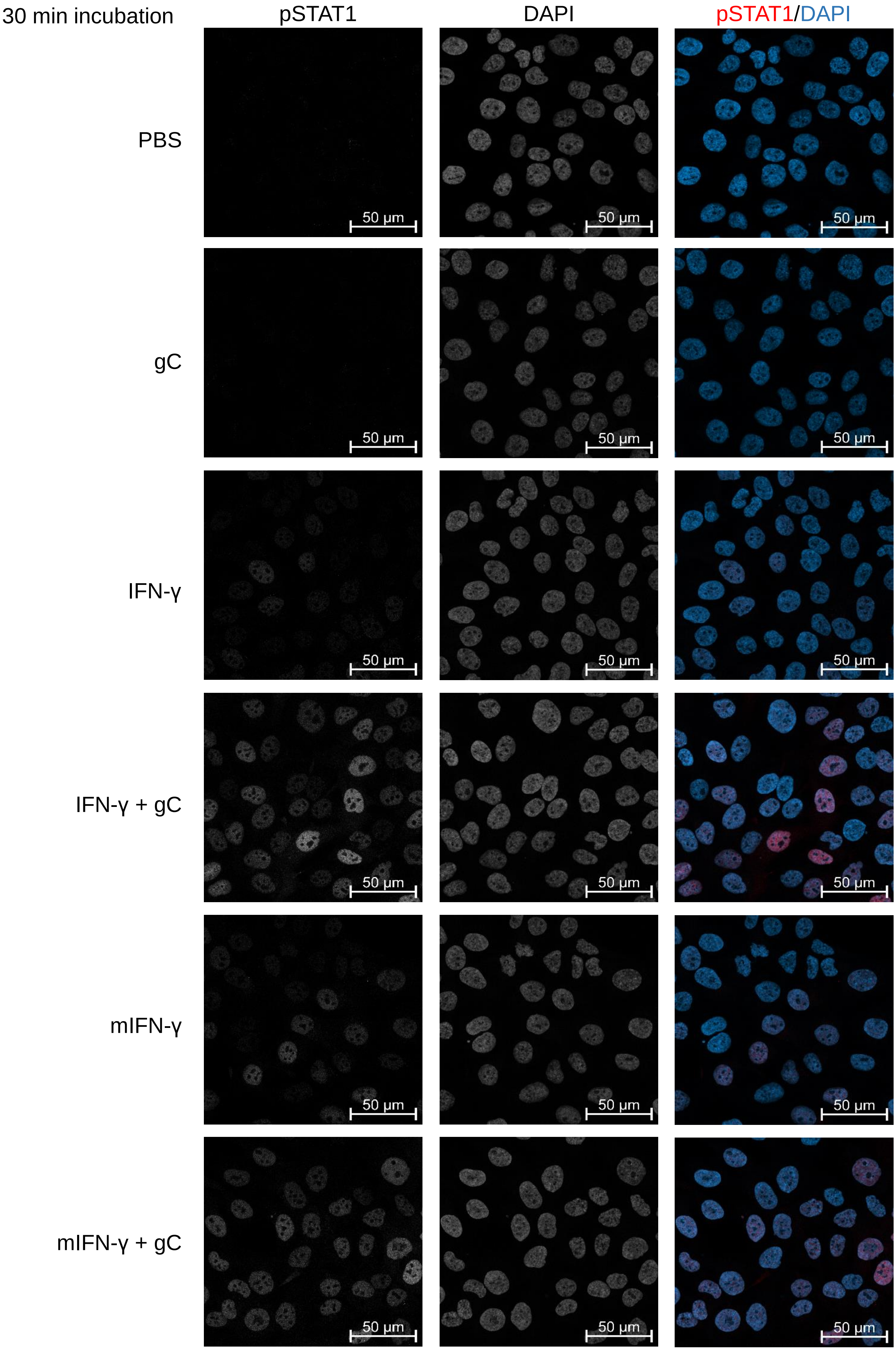

**Supplementary Figure 11. gC$_{S147-V531}$ increases IFN-γ-mediated nuclear translocation of p-STAT1 at 30 minutes post-incubation.** Immunofluorescence images showing p-STAT1 in the cytoplasm and nucleus of HaCaT cells incubated during 30 minutes at 37 °C with PBS, gC$_{S147-V531}$, IFN-γ expressed in bacteria or in mammalian cells (mIFN-γ) or both gC$_{S147-V531}$ and IFN γ. p-STAT1 was detected with a rabbit monoclonal anti-p-STAT1 Y701 antibody and nuclei were labelled with DAPI. The first two columns show the individual channels in grey while the third column shows both p-STAT1 (red) and DAPI (blue). n=2 biological replicates, except for mIFN-γ, which was performed only once. Scale bar 50 µm

# Supplementary Figure 12

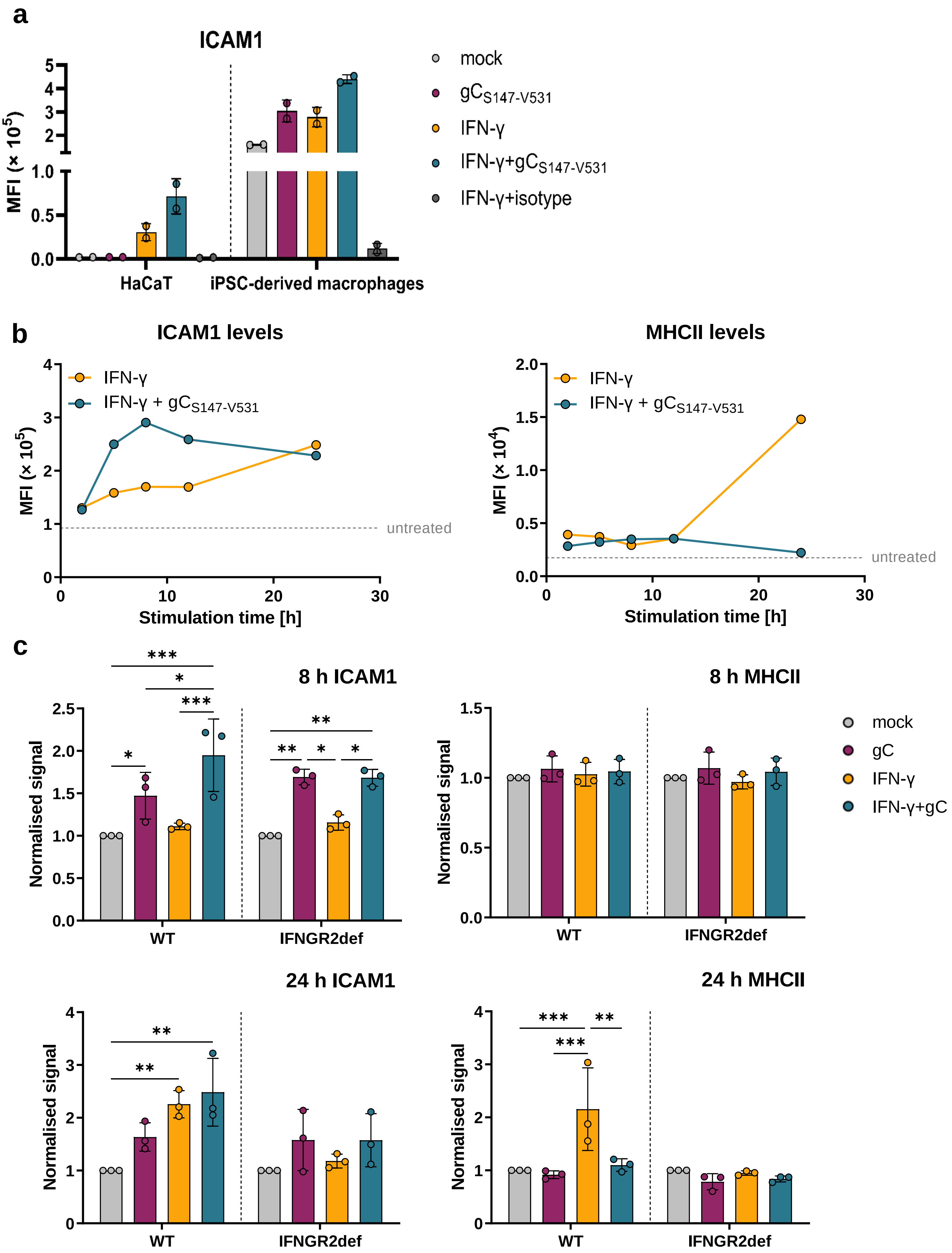

**Supplementary Figure 12. Effect of gC on ICAM1 protein level in iPSC-derived macrophages. (a-c)** HaCaT cells (a) or iPSC-derived macrophages from a healthy donor (wild type, WT) (a-c) and from and IFNGR2-deficient donor (c) were mock-stimulated or stimulated with 5 ng/mL IFN-γ, 300 nM $gC_{S147\text{-}V531}$ or both for the indicated time points and then labelled with antibodies to ICAM1, MHCII, and stained with Zombie-NIR dye. Cells were analyzed by flow cytometry and median fluorescence intensities (MFI) were determined after gating on alive single cells. MFI was normalized to the mock-treated cells for each donor within each experiment. Each circle represents the results of one independent biological replicate (n=2 in biological replicates in a; n=1 biological replicate in b; n=3 in biological replicates in c), bars show mean values ± SD. Two-way ANOVA, followed by Tukey's multiple comparisons was performed (comparison between all treatments within each donor). Non-significant comparisons are not depicted. * = $P$ <0.033; ** = $P$ <0.002; *** = $P$ <0.001.

Supplementary Figure 13

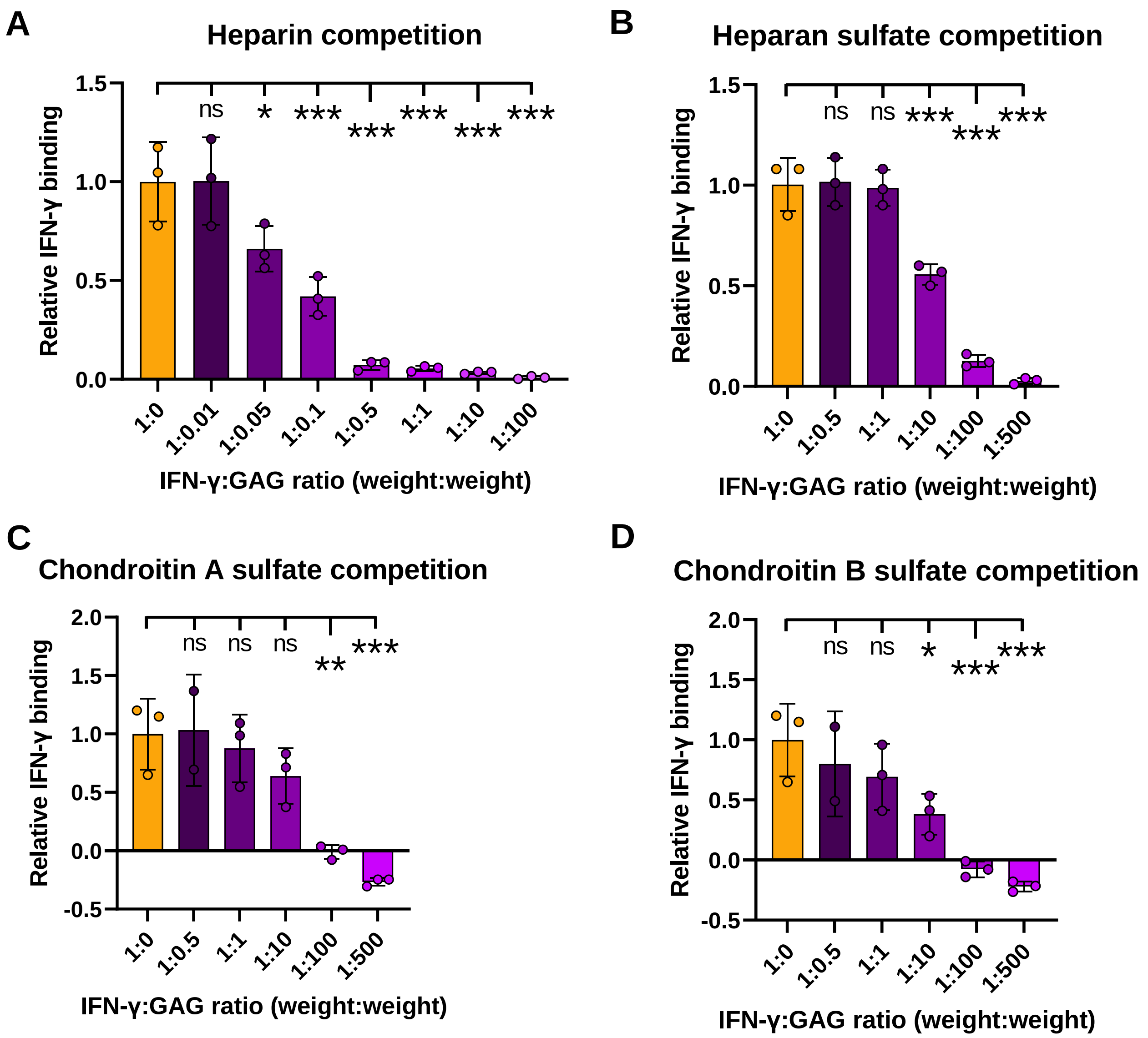

**Supplementary Figure 13. GAGs compete with the gC – IFN-γ interaction. (a-d)** Bar graphs showing the effect of heparin (a), heparan sulphate (b), chondroitin A sulphate (c) and chondroitin B sulphate (d) on binding of IFN-γ to gC. Purified $gC_{S147-V531}$ was immobilized on a CM5 sensor chip (9,800 RU) and IFN-γ was injected at 100 nM either alone or together with increasing amounts of different GAGs (weight ratio). The response levels at the binding report point were normalized to the response obtained for IFN-γ alone and plotted for the different weight ratios. The bars in a-d represent the mean ± SD, the filled circles represent the values from three independent experiments (n=3 biological replicates). One-way ANOVA was performed to test for statistical significance (comparing to control without GAGs), followed by Dunnett's multiple comparisons test. ns = not significant; * = $P$ <0.033; ** = $P$ <0.002; *** = $P$ <0.001

Supplementary Figure 14

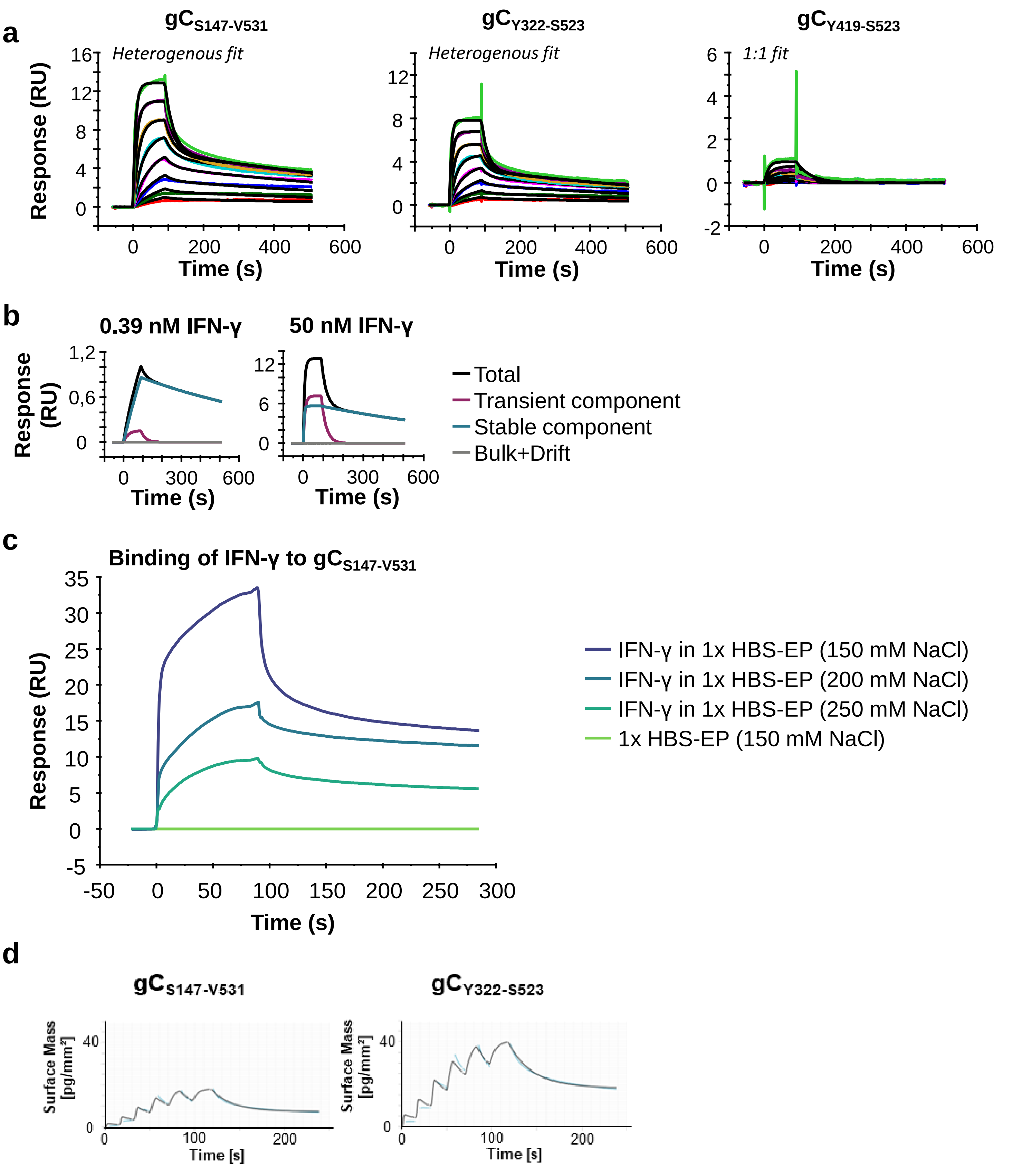

**Supplementary Figure 14. The interaction between gC and IFN-γ contains a stable and a transient component. (a)** Sensorgrams showing the results of assays to determine the kinetics of the interactions between VZV gC constructs and IFN-γ using the Biacore S200. The gC constructs were immobilized on a CM4 sensor chip (877 RU, 399 RU, and 293 RU, respectively) and IFN-γ was injected in a 1:2 dilution series starting at 50 nM with a flow rate of 30 μL/min. Black lines indicate the heterogenous ligand fit or 1:1 binding model (as indicated) determined with the Biacore S200 Evaluation software. **(b)** Sensorgrams showing the contribution of the transient and stable interactions upon injection of a low and high IFN-γ concentration onto the $gC_{S147\text{-}V531}$ chip. **(c)** Sensorgram showing results of binding experiments between $gC_{S147\text{-}V531}$ immobilised on a CM5 sensor chip (877 RU) and IFN-γ. The A-B-A injection scheme of the Biacore S200 system to test different buffer conditions was used. Pre-sample contact time was set to 360 s. The association time was 90 s, followed by 240 s of post-sample contact time. IFN-γ was injected at 100 nM with a flow rate of 30 μL/min. **(d)** Sensorgrams showing the results of assays to determine the kinetics of the interactions between VZV $gC_{S147\text{-}V531}$ and $gC_{Y322\text{-}V523}$ using the Creoptix WAVE system. The gC proteins were immobilised on a DXH chip (567 $pg/mm^3$ and 584 $pg/mm^3$, respectively) and IFN-γ was injected at 200 nM using RAPID in the tight binder mode. Black line indicates the fit using a heterogenous ligand model and traditional fitting.

# Supplementary Figure 15

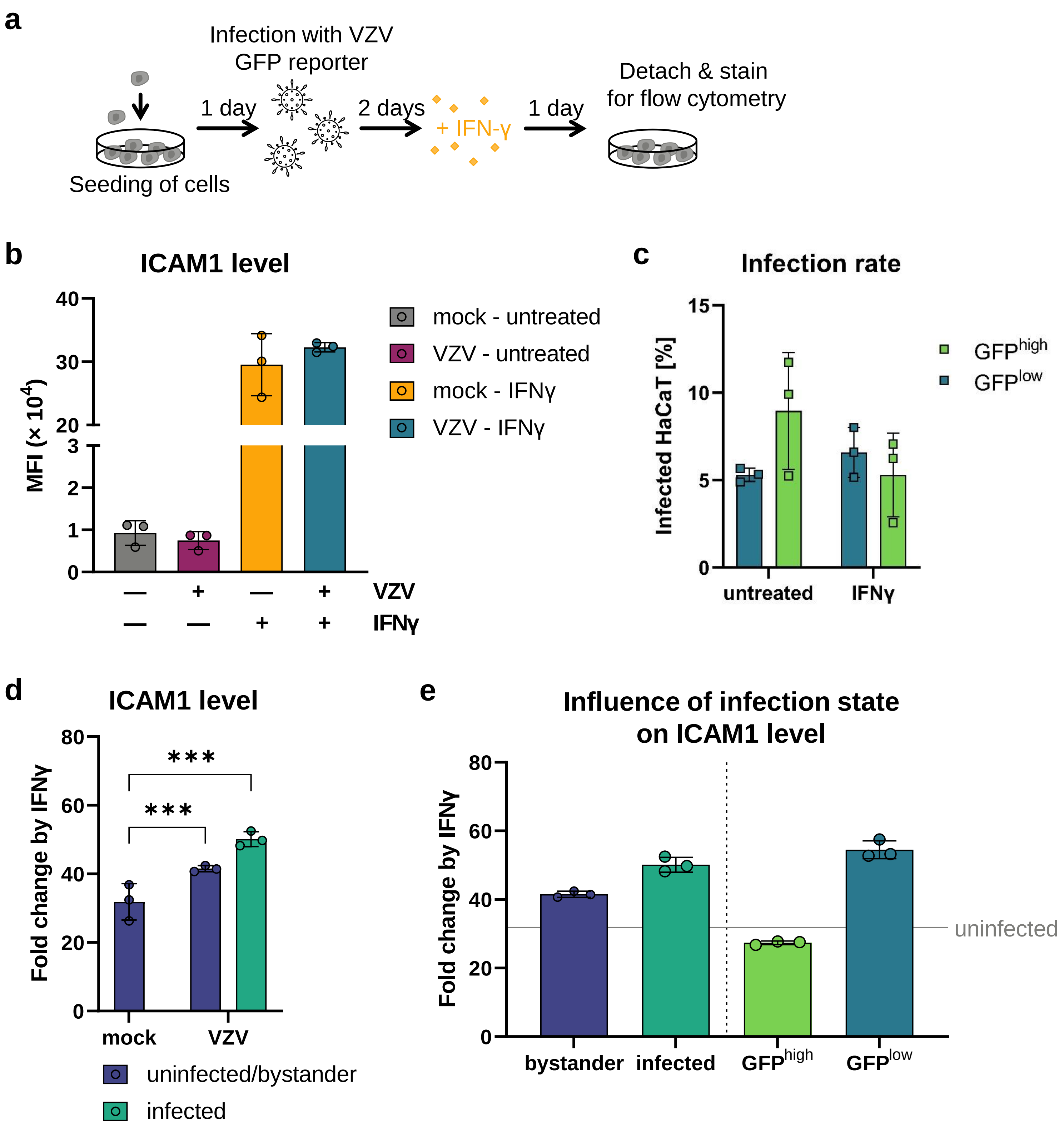

**Supplementary Figure 15. Induction of ICAM1 by IFN-γ is higher in VZV inoculated cultures.** **(a)** Schematic representation of the assay. HaCaT cells were seeded 24 h prior to infection with pOka-Δ57-GFP. 48 h after infection, cells were stimulated with IFN-γ or mock treated. The next day, cells were detached and labelled with anti-ICAM1-APC and stained with Zombie-NIR dye, fixed, and analyzed by flow cytometry. **(b)** Bar chart showing the median fluorescence intensities (MFI) for ICAM1 in the four treatment conditions after gating on alive cells. **(c)** Bar chart showing the percentage of infected HaCaT cells in mock- and IFN-γ-treated cultures after gating on alive cells. The different colors discriminate the proportion of $GFP^{high}$ and $GFP^{low}$ expressing cells. Ordinary two-way ANOVA analysis showed no significant differences. **(d, e)** Bar charts showing the calculated fold-change of ICAM1 levels in mock and VZV-infected cells after gating on uninfected or infected cells (d) and after differentiating between $GFP^{high}$ and $GFP^{low}$ cells (e). As reference in (e) the fold change by IFN-γ of uninfected cells (from d) is indicated with a grey line. The data in b-e was obtained from three independent experiments (n=3 biological replicates). Ordinary two-way ANOVA analysis with main effects only followed by Dunett's multiple comparison was performed (d). Bars in (b-e) represent the mean ± SD and filled circles represent the individual values from three independent experiments. ns = not significant; * = $P$ <0.033; ** = $P$ <0.002; *** = $P$ <0.001.

# Supplementary Figure 16

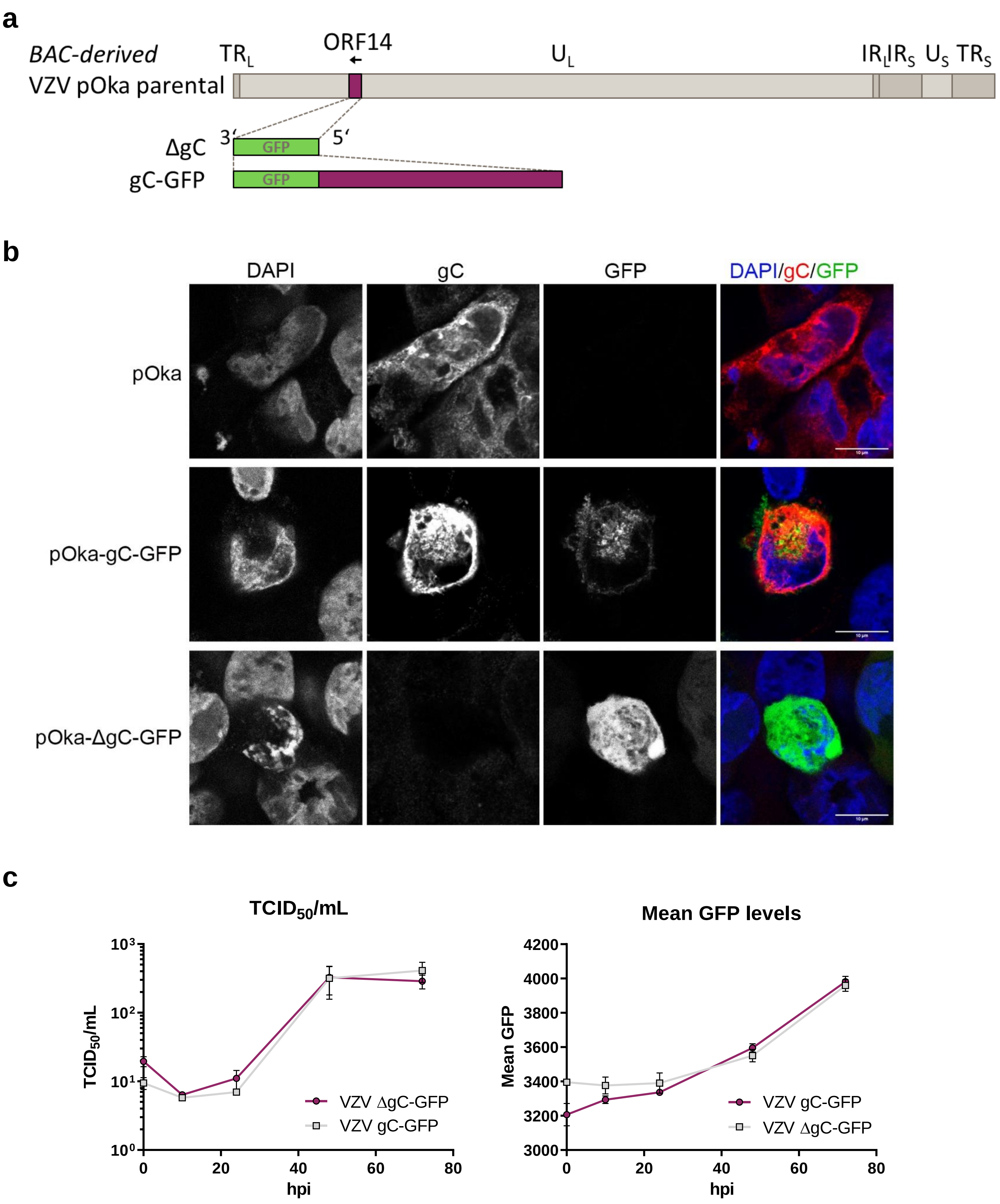

**Supplementary Figure 16. VZV-gC-GFP and VZV-ΔgC-GFP show similar replication kinetics in HaCaT cells. (a)** Schematic representation of the BAC-derived VZV pOka strains. The repeat and unique regions of the parental, BAC-derived, VZV genome are shown. *ORF14*, encoding gC is highlighted in magenta. The arrow above *ORF14* indicates that this gene is located in the reverse DNA strand. Monomeric GFP was inserted instead of the *ORF14* locus and at the 3' end of *ORF14* to generate VZV-ΔgC-GFP and VZV-gC-GFP recombinant viruses, respectively. In both cases, the *ORF14* promoter drives expression of GFP. **(b)** Immunofluorescence images of ARPE-19 cells infected with parental pOka, VZV-gC-GFP and VZV-ΔgC-GFP and labelled with a mouse monoclonal antibody against gC. Nuclei were stained with DAPI. **(c)** HaCaT cells were infected with 100 PFU of the two indicated BAC-derived virus strains. At the different time points post-infection, cells were imaged and collected for titration. Assays were performed in technical triplicates (n=3). Collected cells were titrated and the $TCID_{50}$ was determined on HaCaT cells. $TCID_{50}$ values are plotted over time in the left panel. The right panel shows the mean GFP fluorescence over time measured with the Cytation3 (BioTek). Abbreviations: $TR_l$ = terminal repeat long; $TR_s$ = terminal repeat short; $U_L$ = unique long region; $IR_L$ = internal repeat long; $IR_s$ = internal repeat short; $U_S$ = unique short region; ORF = open reading frame; GFP = green fluorescent protein; $TCID_{50}$ = tissue culture infectious dose 50; hpi = hours post-infection.

Supplementary Figure 17

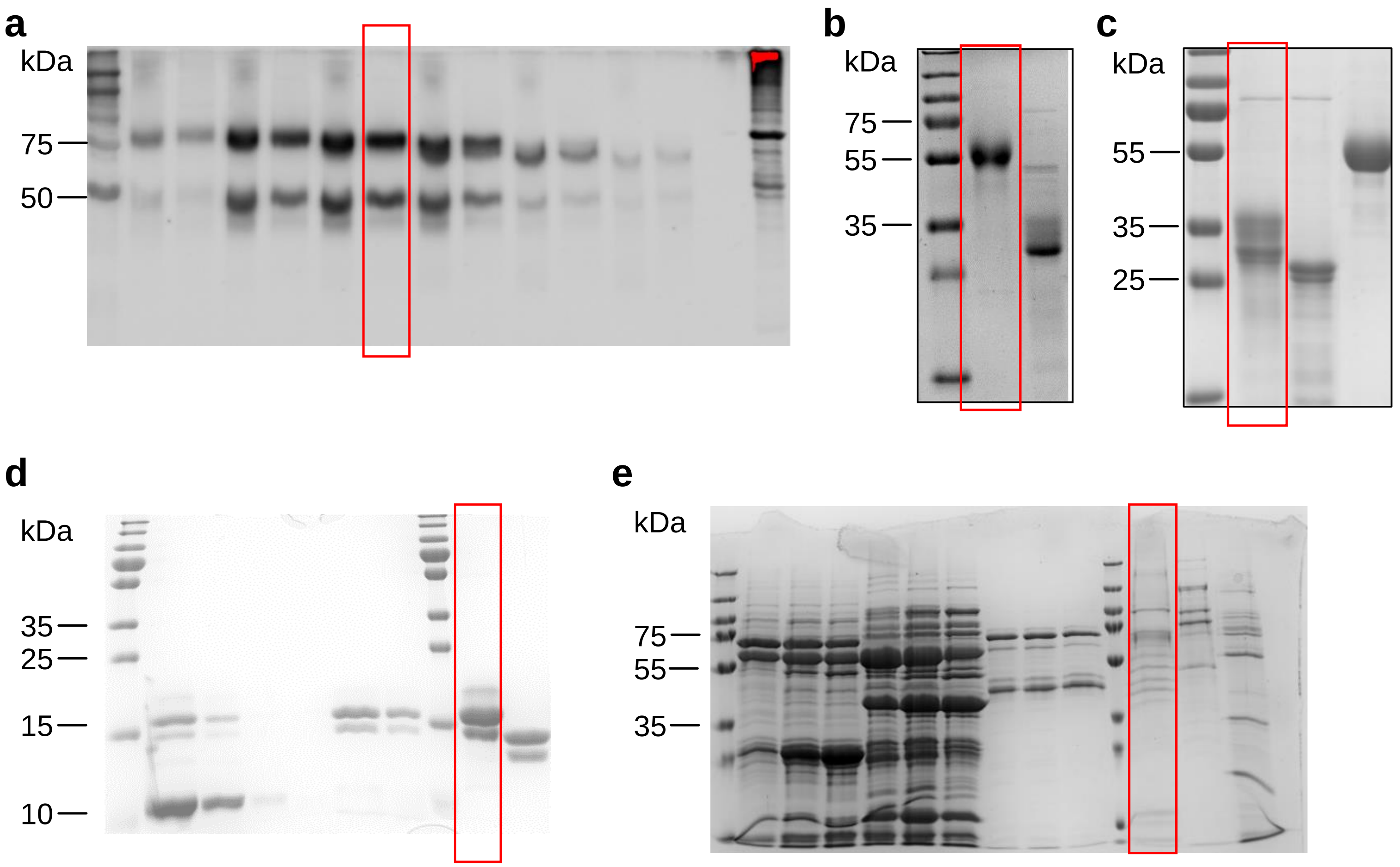

**Supplementary Figure 17. Uncropped gels for Supplementary Figure 1.** Uncropped gels for those shown in Supplementary Figure 1b **(a)**, 1d **(b)**, 1f **(c)**, 1h **(d)** and 1j **(e)**. The lanes shown in Supplementary Figure 1b are surrounded by a red rectangle.

# Supplementary Figure 18

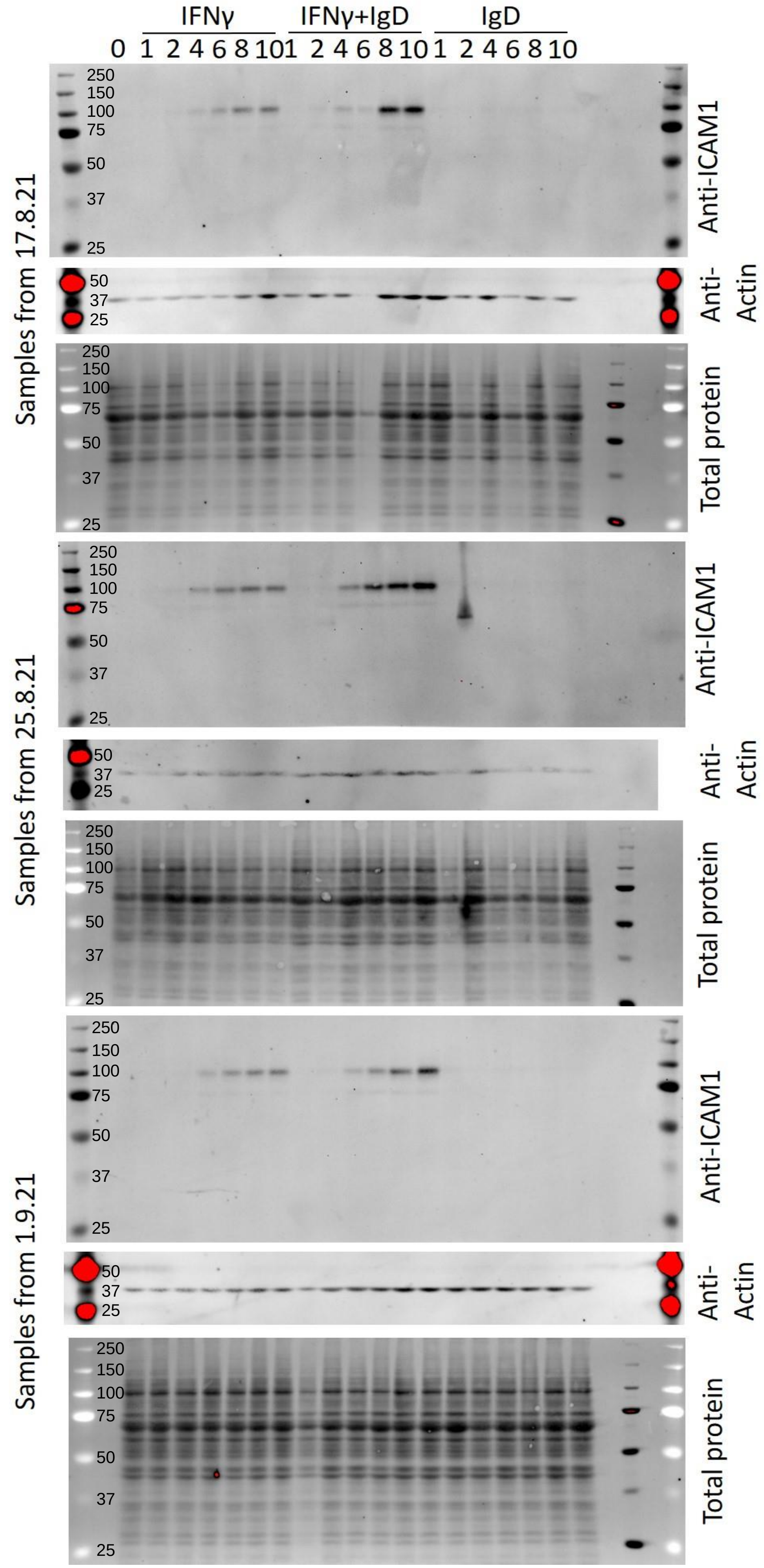

**Supplementary Figure 18. Uncropped western blots for Supplementary Figure 8.** Uncropped western blots from three biological repeats. The three uncropped blots at the bottom correspond to the ones shown in Supplementary Figure 8b.

Supplementary Table 1

| | **Model** | **Ka1 (1/Ms)** | **SE(ka1) (1/Ms)** | **Kd1 (1/s)** | **SE(kd1) (1/s)** | **KD1 (M)** | **Ka2 (1/Ms)** | **SE(ka2) (1/Ms)** | **Kd2 (1/s)** | **SE(kd2) (1/s)** | **KD2 (M)** |
|---|---|---|---|---|---|---|---|---|---|---|---|
| $\mathbf{gC_{S147-V531}}$ | Heterogeneous Ligand | 1,51E+06 | 7,76E+03 | 4,14E-02 | 1,39E-04 | 2,75E-08 | 4,93E+06 | 7,14E+03 | 1,11E-03 | 3,90E-06 | 2,26E-10 |
| $\mathbf{gC_{Y322-S523}}$ | Heterogeneous Ligand | 2,61E+06 | 1,50E+04 | 5,58E-02 | 2,35E-04 | 2,13E-08 | 6,47E+06 | 1,29E+04 | 1,33E-03 | 4,89E-06 | 2,05E-10 |
| $\mathbf{gC_{Y419-S523}}$ | 1:1 Binding | 1,35E+06 | 1,88E+04 | 2,44E-02 | 1,77E-04 | 1,80E-08 | n.a. | n.a. | n.a. | n.a. | n.a. |

Exemplarily kinetic values for the VZV gC – IFN-γ interaction obtained from at least two kinetic assays performed using the Biacore S200. Abbreviations: s = seconds, M = molar (mol/L), Ka = association rate constant, Kd = dissociation rate constant, KD = equilibrium dissociation constant, SE = standard error.

Supplementary Table 2

| | Model | Ka1 (1/Ms) | Kd1 (1/s) | KD1 (M) | Ka2 (1/Ms) | Kd2 (1/s) | KD2 (M) |
|---|---|---|---|---|---|---|---|
| **$gC_{S147-V531}$** | Heterogeneous Ligand | 7,17E+05 | 4,29E-02 | 5,98E-08 | 8,06E+05 | 1,57E-05 | 1,95E-11 |
| **$gC_{Y322-S523}$** | Heterogeneous Ligand | 6,18E+05 | 3,91E-02 | 6,33E-08 | 6,84E+05 | 3,28E-04 | 4,80E-10 |

Exemplarily kinetic values for the VZV gC – IFN-γ interaction obtained from at least three kinetic assays performed using the Creoptix WAVE systems. Abbreviations: s = seconds, M = molar (mol/L), Ka = association rate constant, Kd = dissociation rate constant, KD = equilibrium dissociation constant.

Supplementary Table 3

| Oligo name | Sequence (5’ to 3’) |
|---|---|
| gC$_{P23-V531}$-forward | TATAGATCTCCCACACCCGTAAGTATAACT |
| gC$_{S147-V531}$-forward | TATAGATCTTCACAACCACCTTTTCTA |
| gC-reverse | TATTTAACTAGTAACGGAAAATGTAGTGGC |
| gC$_{Y322-S523}$-forward | CTTTGTTGGCCTCTCGCTCGGGTATCGTCCAAATATTACCGTTGTCG |
| gC$_{Y419-S523}$-forward | CTTTGTTGGCCTCTCGCTCGGGTATTCTGCTGTCGTTACCCC |
| gC$_{RFC}$-reverse | CAACCGGCCTTATCGTCATCGTCAGATGCATCGTAGGTATAAACGG |

Oligonucleotides employed for cloning the different gC constructs.

Supplementary Table 4

| Oligo name | Sequence (5’ to 3’) |
|---|---|
| Actin-fwd | CTTCGCGGGCGACGAT |
| Actin-rev | CCACATAGGAATCCTTCTGACC |
| ICAM1-fwd | GTATGAACTGAGCAATGTGCAAG |
| ICAM1-rev | GTTCCACCCGTTCTGGAGTC |
| CXCL8-fwd | ACTGAGAGTGATTGAGAGTGGAC |
| CXCL8-rev | AACCCTCTGCACCCAGTTTTC |
| CXCL9-fwd | GGTGTTCTTTTCCTCTTGGGC |
| CXCL9-rev | AACAGCGACCCTTTCTCACT |
| CXCL10-fwd | GTGGCATTCAAGGAGTACCTC |
| CXCL10-rev | TGATGGCCTTCGATTCTGGATT |
| CXCL11-fwd | GAGTGTGAAGGGCATGGCTA |
| CXCL11-rev | ACATGGGGAAGCCTTGAACA |
| IL4I1-fwd | GCCAAGACCCCTTCGAGAAAT |
| IL4I1-rev | CCGATCCTGTTATCTGCCTCC |

Oligonucleotides employed for qPCR

## Supplementary Materials and Methods

### Bacteria

*Escherichia coli* (*E. coli*) DH5α was grown in LB medium shaking at 200 – 220 rpm or on LB agar at 37 °C. This strain was used for general cloning purposes. The VZV BAC-containing *E. coli* strain GS1783 was cultured in LB medium containing 17 µg/mL chloramphenicol shaking at 200-220 rpm or on LB agar containing 17 µg/mL chloramphenicol at 30-32 °C.

### Cell culture

A549 is an epithelial cell line isolated from lung tissue of a caucasian male with lung cancer and was provided by Thomas Pietschmann (Twincore, Hannover, Germany). HaCaT cells are *in vitro* spontaneously transformed keratinocytes derived from histologically normal skin from a caucasian male. These cells were provided by Beate Sodeik (Institute of Virology, MHH, Germany). Jurkat E6.1 originate from a male patient with acute T cell leukemia and were kindly provided by Martin Messerle (Institute of Virology, MHH, Germany). Jurkat LFA-1 KO cells were a gift from Carsten Münk (Düsseldorf University Hospital, Germany) and have been described previously[1]. MeWo cells are derived from a male patient suffering malignant melanoma and were purchased from ATCC (HTB-65™). Human retinal pigment epithelial cell line ARPE-19 (ATCC-CRL-2302) was cultured in Dulbecco’s modified Eagle medium/Nutrient Mixture F-12 Ham (D/F-12; SIGMA).

Peripheral blood mononuclear cells (PBMCs) from anonymised healthy blood donors were isolated using standard Ficoll-density centrifugation methods. Isolated PBMCs were washed twice, and remaining erythrocytes were lysed with ACK lysing buffer (Lonza). The PBMCs were frozen in 90% FBS and 10% DMSO and freshly thawed before experiments.

Primary cultures of normal human keratinocytes (NHEK) were prepared from surgical residuals of foreskin obtained from anonymized children undergoing surgery. Preparation of keratinocytes was done as before [2]. Skin specimens were cut into small pieces and incubated overnight in dispase II (2.4 U per mL, Roche Applied Science) at 41°C. Epidermis was detached from the dermis with fine forceps and incubated in Hank's solution with 0.25% trypsin (Sigma-Aldrich) for 20 min at 371C. Trypsin activity was stopped by addition of FCS (Life Technolologies, Karlsruhe, Germany). The cell suspension was passed through a 40 mm sterile gauze. The obtained keratinocytes were washed twice and single-cell suspension of keratinocytes was cultured in serum-free keratinocytes growth medium (Keratinocyte Growth Medium 2 Kit; PromoCell GmbH, Heidelberg, Germany). The purity of keratinocytes was verified by the expression of the epithelial marker cytokeratin (mouse anti-human cytokeratin antibody, clone: MNF-116, DakoCytomation, Hamburg, Germany) and the fibroblast-specific marker ASO2 (CD90, Dianova GmbH, Hamburg, Germany). All cells (more than 95%) were found to be uniformly positive for cytokeratin but not for CD90.

All mammalian cells were cultured at 37 °C with 5% $CO_2$ in a humidified incubator. A549, HaCaT and Mewo cells were cultured in DMEM (Gibco™ #41966-052), supplemented with 8% heat-inactivated FBS (Sigma #F7524), 1× L-glutamine (Cytogen #04-80100) and 1× penicillin/streptomycin (Cytogen #06-07100). Jurkat E6.1 and Jurkat LFA-KO cells were cultured in RPMI1640 (Gibco™ #21875-034), supplemented with 8% heat-inactivated FBS, 1× L-glutamine and 1× penicillin/streptomycin, with the addition of 2 µg/mL puromycin (Invivogen #ant-pr) for the Jurkat LFA-KO cells. PBMCs were maintained in RPMI1640 supplemented with 10% heat-inactivated FBS, 1× L-glutamine, 1× sodium pyruvate (Gibco™ #11360-070) and 1× penicillin/streptomycin.

Schneider’s *Drosophila melanogaster* Line 2 (S2) cells (ATCC No.: CRL-1963) are derived from embryonic tissue of the fruit fly *Drosophila melanogaster* and were used for protein production after stable transfection. The cell line was purchased from Thermo Scientific. These cells were grown in Schneider’s *Drosophila* medium (Gibco) supplemented with heat-inactivated 10% FBS and 1× penicillin/streptomycin (Gibco) at 28 °C with normal atmospheric conditions in a semi-adherent manner for maintenance and transfection. After transfection, 8 µg/mL puromycin was added to the media for selection during maintenance. For protein production, this semi-adherent cell line was grown in suspension by shaking at 70 rpm in baffled Erlenmeyer flasks using Insect XPRESS medium (Lonza).

For the generation of healthy iPSC-derived macrophages, both hCD34iPSC16 (MHHi015-A: https://hpscreg.eu/cell-line/MHHi015-A) and the GMP research grade LiPSC-GR1.1 iPSC line (GMP-grade, Lonza, Basel, Switzerland) were used. The patient-specific iPSC line derived from a patient harbouring the *IFNGR2* c.705C>A mutation (citation: 10.3390/cells9020483) was used for the generation of the IFNGR2-deficient macrophages. iPSC-derived macrophages were generated as described previously[3, 4]. Briefly, upon expansion of the iPSC cells, bFGF was omitted from the culture medium and embryoid bodies (Ebs) were formed on an orbital shaker at 80 rpm. On day 5, the properly developed Ebs were selected and transferred into an adherent 6-well plate with differentiation medium (X-vivo; Lonza) supplemented with 1% penicillin/streptomycin, 1 mM L-glutamine, 0.05 mM β-mercaptoethanol, 50 ng/mL M-CSF (Peprotech), and 25 ng/mL IL-3 (Peprotech). In case of feeder-free iPSC culture conditions, the protocol was modified accordingly [5, 6] and to promote hematopoietic differentiation the mesoderm priming medium was supplemented with the cytokines BMP4, SCF and VEGF. The medium of the differentiation cultures was replaced once weekly and the generated macrophages were harvested and terminally

differentiated in RMPI medium supplemented with 10 % FCS (Sigma Aldrich), 1 % penicillin/streptomycin and 50 ng/mL M-CSF (Peprotech) (0.1 x $10^6$ cells/well a 48-well plate).

**Preparation of virus stocks**

Non-infected cells were seeded the day prior to infection to about 75% confluency. For infection, an inoculation rate of one infected cell to 5-7 uninfected cells was used in media with 2% FBS. The cell-associated virus inoculum was left on the cells in case the same cell line was used for virus production. Otherwise, the inoculum was removed after 2 – 3 h and the cells were washed to remove any contaminating cell types. Infected cells were frozen in medium with 20% FBS and 10% DMSO and used as inoculum after thawing and removal of the DMSO-containing freezing medium.

**VZV replication kinetics**

HaCaT cells were seeded one day prior to infection with a density of 9 × $10^4$ cells/well of a 24-well plate. Cells were subconfluent for infection with 100 PFU/well in 250 µL infection medium (growth medium with only 2% heat-inactivated FCS) for 2 h at 37 °C, 5% CO2, in a humidified incubator. Inoculum was washed off and replaced by infection medium. Samples were harvested at 0, 10, 24, 48 and 72 hours post-infection by replacing the medium with 1× DPBS, followed by imaging at the Cytation3 (BioTek). Then, cells were detached using trypsin/EDTA, frozen in the respective growth medium described in the '"Cell culture" section, supplemented with 20% FCS and 10% DMSO, and stored at -80 °C until titration of the samples. The experiment was performed with triplicates.

**End point dilution assays**

End point dilution assays were performed to determine the tissue culture infectious dose 50 ($TCID_{50}$) values. HaCaT cells were used for titration of viruses. Cell-associated virus from HaCaT cells was thawed quickly in a 37 °C water bath, followed by washing with infection medium to remove DMSO. A 1:5 dilution series of the virus was prepared in infection medium, with at least 4 replicas per virus stock. The medium of HaCat cells was discarded and replaced by the diluted virus followed by incubation for 5 to 6 days. The infected wells per dilution step were counted and the $TCID_{50}$/mL and PFU/mL were calculated using the $TCID_{50}$ calculator provided online by Marco Binder (Dept. Infectious Diseases, Molecular Virology, Heidelberg University, Germany), which is based on the Spearman & Kärber algorithm. The PFU/mL was calculated by multiplying the $TCID_{50}$/mL by 0.69.

**Detection of phosphorylated STAT1 (p-STAT1).**

HaCaT cells were seeded on 24-well plates (3.0 x $10^5$ cells/well) and incubated overnight at 37 °C and 5% $CO_2$. On the following day, confluent cell monolayers were washed twice with PBS, starved for 5 hours in DMEM containing 0.1% BSA (Sigma), 100 units/mL penicillin, 100 µg/mL streptomycin (Pen/Strep; Cytogen) and 2 mM L-glutamine (Cytogen) and stimulated for 10 or 30 min with 5 ng/mL IFN-γ, 300 nM gCS147-V531 or both (pre-incubated during 30 minutes). Following treatment, cells were washed with PBS, scraped off wells and pelleted at 2,500 x g and 4 °C for 5 min. HaCaT cells were lysed in RIPA buffer supplemented with 1x Halt protease/phosphatase inhibitor cocktail (Thermo Fisher) followed by SDS-PAGE and western blotting using a rabbit monoclonal antibody against phosphorylated Y701 in human STAT1 (Cell Signaling Technology #9167S) and a goat anti-rabbit immunoglobulins secondary antibody with horseradish peroxidase (HRP; Dako #P0448). Signal intensities from phosphorylated STAT1 (p-STAT1) labeling were

quantified with Image J[7] and ratios relative to total protein content per lane, as determined by TCE staining, were plotted using GraphPad Prism.

For nuclear p-STAT1 detection by immunofluorescence, glass coverslips were placed on 24-well plates and treated with 0.1 mg/mL poly-L-lysine (Sigma) for 2 hours at 37 °C, prior to the seeding of 1.5 x $10^5$ HaCaT cells per well. Following overnight incubation, cells were washed twice with PBS and starved for 5 hours prior to stimulation for either 10 or 30 min with 5 ng/mL non-glycosylated IFN-γ (Peprotech; produced in *E. coli*), 300 nM $gC_{S147-V531}$ or both, with mixes being pre-incubated for 30 min at 37 °C. Alternatively, glycosylated IFN-γ (R&D; expressed in HEK293, termed mammalian IFN-γ, mIFN-γ) was used. Then, immunofluorescence was performed (see “immunofluorescence” section below).

**SDS-PAGE and western blot**

Protein samples were separated by SDS-PAGE in gels containing 1% TCE[8]. After the run, gels were transferred into VE-water and imaged after 3× 45 s activation using the stain-free gel settings at a ChemiDoc™ MP Imaging System. Alternatively, Coomassie staining was performed to visualize total protein. The proteins were then transferred onto nitrocellulose membranes (PALL via VWR #66485) and imaged for total protein in VE-water using the ChemiDoc stain-free settings, and then blocked with 5% milk in PBS-T (0.1% Tween20) for 1 h at RT. Subsequently, primary antibodies (anti-ICAM1, Cell Signalling Technologies #4915S and anti-β-actin, Thermo Scientific #MA 1-140; rabbit monoclonal anti-phosphorylated Y701 in human STAT1 (Cell Signaling #91675)) in 5% (w/v) milk in DPBS-T were added and incubated overnight at 4 °C. After washing 4 times, the membrane was incubated with secondary antibodies (Goat αMurine IgG IRDye680RD, LI-COR #925-68070 and Goat αRabbit IgG IRDye800CW, LI-COR #925-32211) in 2.5% (w/v) milk in DPBS-T for 1 h at RT in the dark. Finally, the

membrane was washed again 3 times with DPBS-T and was transferred into 1× DPBS for imaging at the ChemiDoc™ MP. Quantification was performed using the band and lane tool of Image Lab™ 6.0.1 software. For detection of p-STAT1, blots were washed with TBS and blocked with 5% BSA in TBS-T (0.1% Tween20) instead. For staining, primary (rabbit monoclonal anti-phosphorylated Y701 in human STAT1, Cell Signaling Technology #9167S) and secondary (polyclonal goat anti-rabbit immunoglobulins with HRP, Dako #P0448) antibodies were incubated in blocking buffer, and signal was developed with the SuperSignal™ West Pico PLUS Chemiluminescent Substrate (Thermo Fisher) prior to detection at the ChemiDoc™ MP.

**Immunofluorescence**

To detect p-STAT1, cells were washed twice with ice-cold PBS, fixed in 4% PFA at 4 °C for 10 min and washed again. Cells were permeabilized with ice-cold methanol for 10 min at -20 °C and rehydrated for 10 min at room temperature with PBS. Blocking was carried out with a 3% BSA and 0.3% Triton X-100 in PBS solution for 1 hour at room temperature with gentle rocking of plates. Later, rabbit monoclonal anti-p-STAT1 antibody (Cell Signaling Technology #91675) was incubated at 1:400 overnight at 4 °C, followed by Alexa Fluor 555-conjugated donkey anti-rabbit IgG secondary antibody (Thermo Fisher #A-31572) at 1:1000 in blocking buffer for 1 hour at room temperature. After wash (twice with PBS and once with water), coverslips were mounted with ProLong Gold Antifade medium (Thermo Fisher) for 24 h at room temperature and analyzed by the Zeiss LSM 980 with Airyscan 2 (Zeiss). Signal intensities from nuclear p-STAT1 staining were quantified through a pipeline on CellProfiler, being plotted afterwards on GraphPad Prism. Samples from different time points (10 and 30 min) were subjected to distinct laser settings in the microscope to allow for a more discriminative analysis and quantification within experimental stimulatory conditions.

To detect gC in infected cells, ARPE-19 cells were infected with cell-associated VZV pOKA-gC-GFP, pOka-ΔgC-GFP or VZV-pOKA WT (parental BAC) at an MOI of 0.05. At 4 dpi, cells were washed with PBS and fixed with 4% paraformaldehyde at room temperature for 15 minutes. Then, cells were washed 3 times with PBS and incubated in permeabilizing and blocking solution (PBS containing 0.5 % Triton X-100, 5 % BSA) for 1 hour. Then, cells were labelled with the primary antibody mouse anti-ORF14 (clone VZ 14.12, CapRi, Croatia), diluted 1:200 in PBS containing 0.1% Triton X-100, 1% BSA at 4 °C overnight. Cells were then washed 3 times with PBST (PBS containing 0.1% Tween 20), incubated 1 hour at room temperature with DAPI and conjugated secondary antibody anti-mouse IgG Alexa Fluor 555 (Life Technologies, 1:1000). Finally, the cells were washed 3 times with PBST and mounted onto glass slides with Prolong Gold Antifade Mountant (Thermo Fisher). Images were obtained using a Zeiss observer Z1 inverted microscope.

**Illumina-Sequencing**

Between 283 ng and 330 ng DNA were used as input in the NEB Ultra II FS Library preparation kit (New England Biolabs, Massachusetts, USA). The fragmentation time was set to 7.30 min, targeting an insert size of 450-500 bp. The libraries were size selected, using 25 µl and 10 µl SPRI Beads and amplified 3 cycles with NEB 2 x 8 bp UDIs. The resulting libraries were pooled and loaded at a concentration of 12 pM on a MiSeq 600v3 Sequencing Kit.

**Nanopore Sequencing**

200 ng of each sample was used as input for the Oxford Nanopore Rapid Barcoding Kit v14 (Oxford Nanopore Technologies, Oxford, UK), following the manufacturers protocol. The pooled libraries were loaded on a MinION Flow Cell (R10.4.1) and sequenced for 48 hours. Basecalling and demultiplexing were performed on an HPC cluster and A100 card, using Guppy v6.5.7 with the dna_r10.4.1_e8.2_400bps_sup model and adapter trimming activated in the guppy_barcoder.

**Sequence assembly**

Illumina and nanopore reads were combined for a *de novo*-hybrid-assembly approach, using a wrapper script. The Illumina reads were trimmed using fastp v0.20.1 (-q 20)[9] and filtered against the host genome by alignment with bowtie2 v2.4.2[10] with only unaligned reads retained for downstream processing. The nanopore reads were adapter trimmed with porechop v0.2.4[11] and quality filtered, using filtlong v0.2.1 (https://github.com/rrwick/Filtlong; --keep percent 90, -min_length 1000). For host filtering, the nanopore reads were classified with Kraken 2 v2.1.1[12] against a host database, made from the human genome hg38, using the included tool Kraken2-build. All unclassified reads were extracted and used for the *de novo* assembly of the viral genome. The assembly of the trimmed reads were performed by several assemblers. For hybrid assembly Unicycler v0.4.8[13] (--no-rotate, --mode bold) was used. For nanopore-read only assembly, Flye v2.9.2-b1786[14], Canu v2.2[15], Raven[16] v1.8.1, Redbean (wtdbg2)[17] and NextDenovo v2.5.2[18] were used. The resulting contigs underwent several polishing stages. First, three rounds of Minimap2[19] v2.26-r1175 / Racon[20] v1.5.0 polishing were applied, followed by one round of Medaka v1.8.0 polishing (https://github.com/nanoporetech/medaka), using the corresponding basecalling model. As a last step, the raw Illumina reads were strictly trimmed with

fastp (--cut-right, --cut_right_mean_quality 20, -l 30) und used for three rounds of polishing with bowtie2 and Pilon v1.24[21] (--mindepth 20, --fix all). After transferring the polished contigs into Geneious Prime 2023 (https://www.geneious.com/), the contigs were annotated and the best result was selected for finalizing the genome. Where missing sequences at the termini were observed, the sequences were reconstructed using the sequences from other contigs or by using the consensus of overhanging long reads. To control the results, the Illumina and Nanopore reads were mapped back to the final genome and the mappings were checked for breaks or mismappings. To identify problematic sites, a variant calling was performed in Geneious Prime, using the strictly trimmed Illumina reads (Maximum Variant Frequency 0.0025, Minimum Strand_Bias P-value 10e-5 when exceeding 65% bias). The variant sites >50% were inspected and manually corrected, if necessary and not based on mapping errors. The annotations were transferred in Geneious Prime, using the function “Annotate from…” and the VZV strain Dumas reference genome (NC_001348) as a template.

## References:


1. Hain A*, et al.* IL-2 Inducible Kinase ITK is Critical for HIV-1 Infection of Jurkat T-cells. *Sci Rep* **8**, 3217 (2018).

2. Wittmann M, Purwar R, Hartmann C, Gutzmer R, Werfel T. Human keratinocytes respond to interleukin-18: implication for the course of chronic inflammatory skin diseases. *J Invest Dermatol* **124**, 1225-1233 (2005).

3. Lachmann N*, et al.* Large-scale hematopoietic differentiation of human induced pluripotent stem cells provides granulocytes or macrophages for cell replacement therapies. *Stem Cell Reports* **4**, 282-296 (2015).

4. Ackermann M*, et al.* Continuous human iPSC-macrophage mass production by suspension culture in stirred tank bioreactors. *Nat Protoc* **17**, 513-539 (2022).

5. Buchrieser J, James W, Moore MD. Human Induced Pluripotent Stem Cell-Derived Macrophages Share Ontogeny with MYB-Independent Tissue-Resident Macrophages. *Stem Cell Reports* **8**, 334-345 (2017).

6. Gutbier S*, et al.* Large-Scale Production of Human iPSC-Derived Macrophages for Drug Screening. *Int J Mol Sci* **21**, (2020).

7. Schneider CA, Rasband WS, Eliceiri KW. NIH Image to ImageJ: 25 years of image analysis. *Nat Methods* **9**, 671-675 (2012).

8. Ladner CL, Yang J, Turner RJ, Edwards RA. Visible fluorescent detection of proteins in polyacrylamide gels without staining. *Anal Biochem* **326**, 13-20 (2004).

9. Chen S, Zhou Y, Chen Y, Gu J. fastp: an ultra-fast all-in-one FASTQ preprocessor. *Bioinformatics* **34**, i884-i890 (2018).

10. Langmead B, Salzberg SL. Fast gapped-read alignment with Bowtie 2. *Nat Methods* **9**, 357-359 (2012).

11. Wick RR, Judd LM, Gorrie CL, Holt KE. Completing bacterial genome assemblies with multiplex MinION sequencing. *Microb Genom* **3**, e000132 (2017).

12. Wood DE, Lu J, Langmead B. Improved metagenomic analysis with Kraken 2. *Genome Biol* **20**, 257 (2019).

13. Wick RR, Judd LM, Gorrie CL, Holt KE. Unicycler: Resolving bacterial genome assemblies from short and long sequencing reads. *PLoS Comput Biol* **13**, e1005595 (2017).

14. Kolmogorov M, Yuan J, Lin Y, Pevzner PA. Assembly of long, error-prone reads using repeat graphs. *Nat Biotechnol* **37**, 540-546 (2019).

15. Koren S, Walenz BP, Berlin K, Miller JR, Bergman NH, Phillippy AM. Canu: scalable and accurate long-read assembly via adaptive k-mer weighting and repeat separation. *Genome Res* **27**, 722-736 (2017).

16. Vaser R, Sikic M. Time- and memory-efficient genome assembly with Raven. *Nat Comput Sci* **1**, 332-336 (2021).

17. Ruan J, Li H. Fast and accurate long-read assembly with wtdbg2. *Nat Methods* **17**, 155-158 (2020).

18. Hu J*, et al.* An efficient error correction and accurate assembly tool for noisy long reads. *bioRxiv*, 2023.2003.2009.531669 (2023).

19. Li H. Minimap2: pairwise alignment for nucleotide sequences. *Bioinformatics* **34**, 3094-3100 (2018).

20. Vaser R, Sovic I, Nagarajan N, Sikic M. Fast and accurate de novo genome assembly from long uncorrected reads. *Genome Res* **27**, 737-746 (2017).

21. Walker BJ*, et al.* Pilon: an integrated tool for comprehensive microbial variant detection and genome assembly improvement. *PLoS One* **9**, e112963 (2014).

# Gating strategy for Figure 3a,b

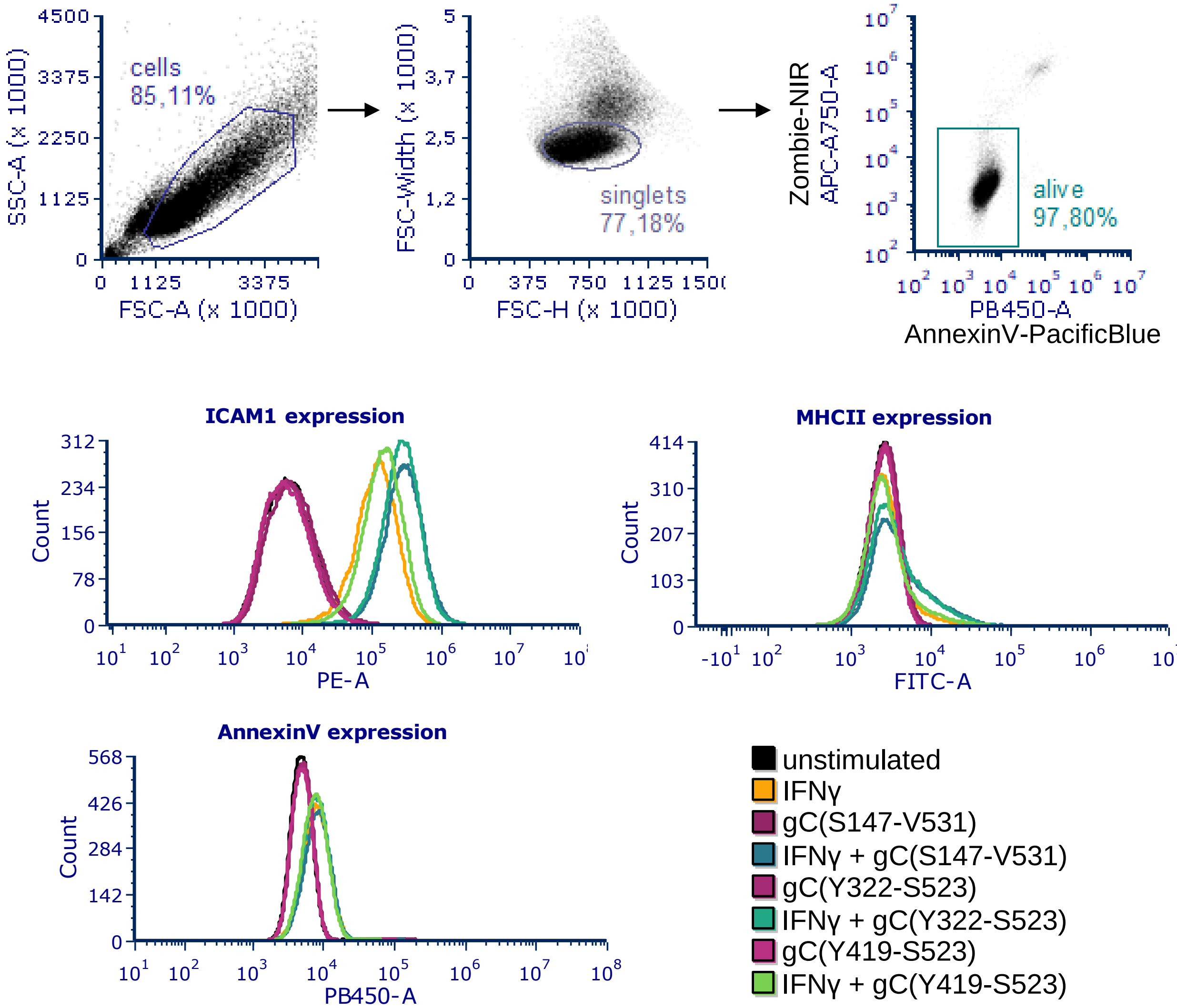


# Gating strategy for Figure 3c

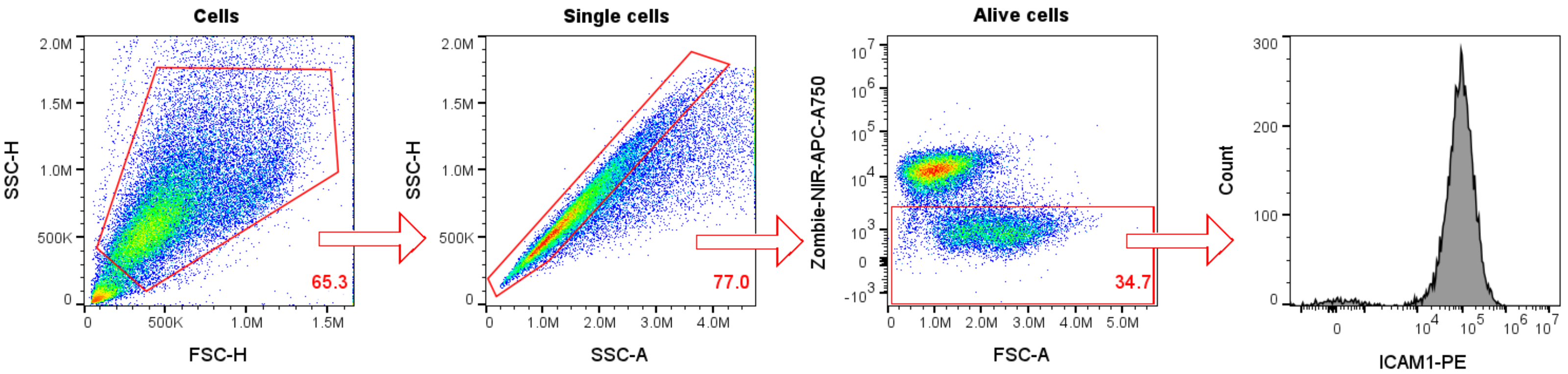

# Gating strategy for Figure 5e

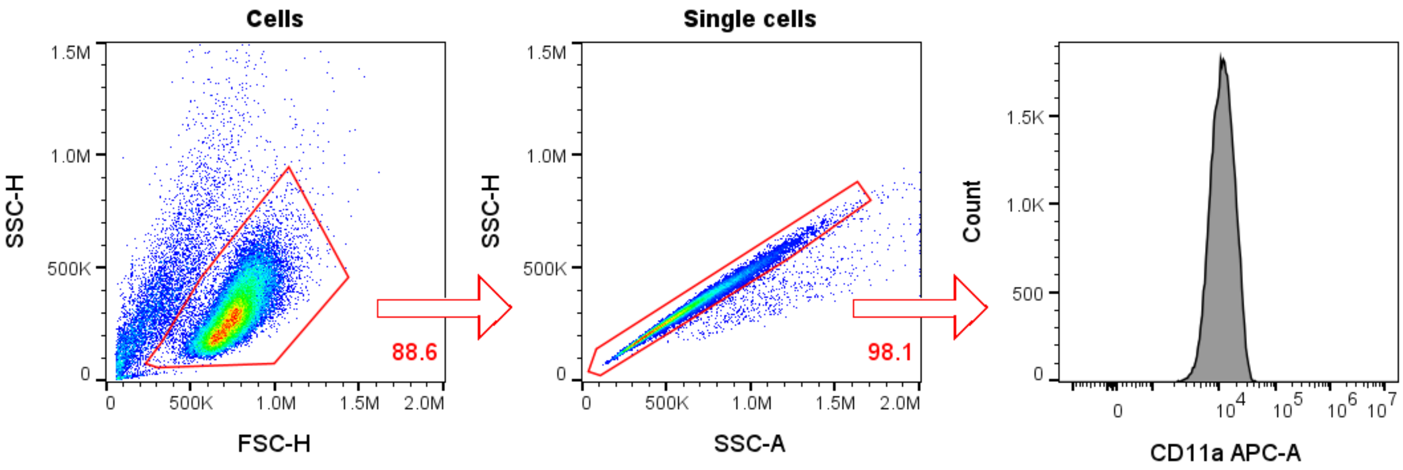


Gating strategy to detect LFA-1 (CD11a) expression in Jurkat cells

# Gating strategy for Figure 7

## Gating strategy

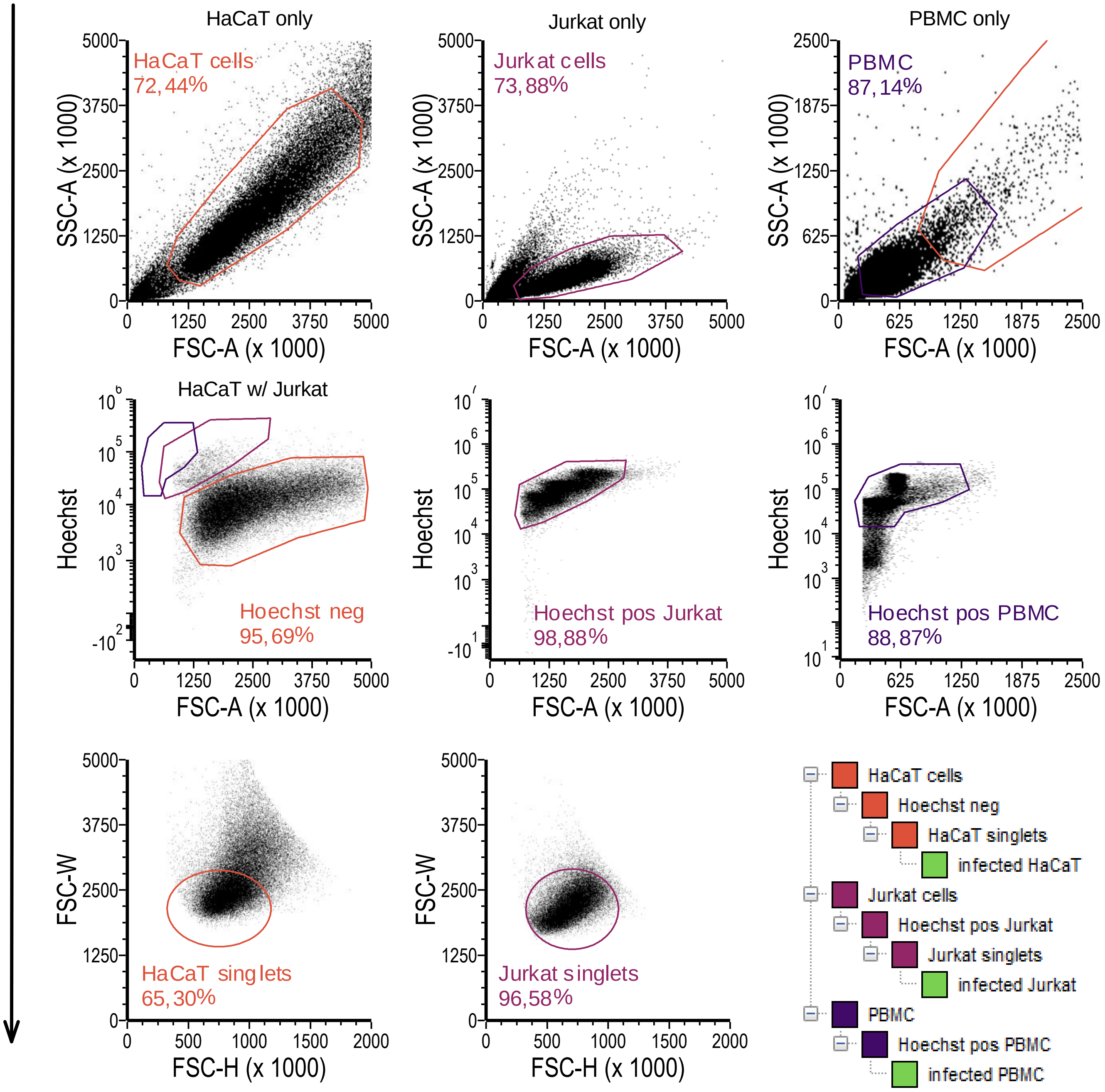

# Dot plots for Figure 3a,b

## Representative dot plots

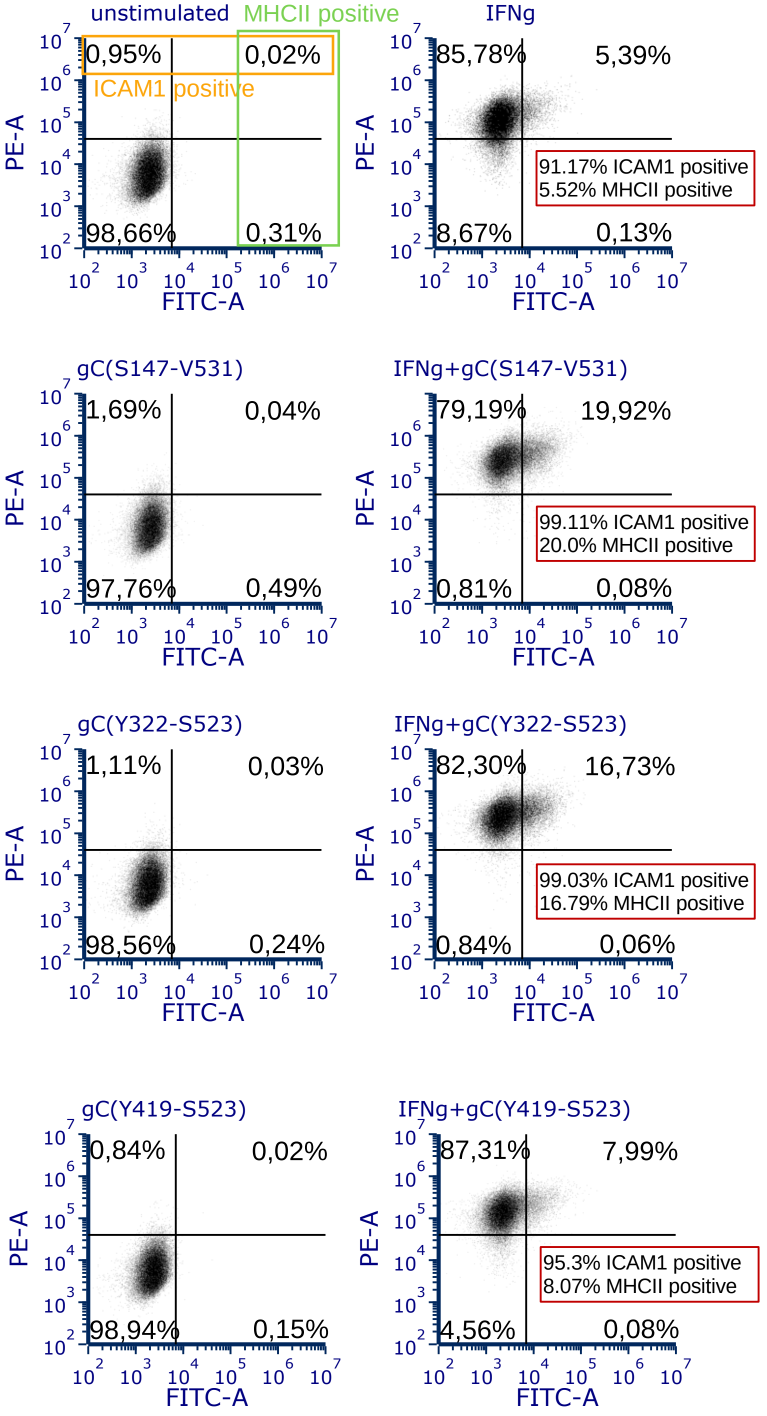


## Representative histograms

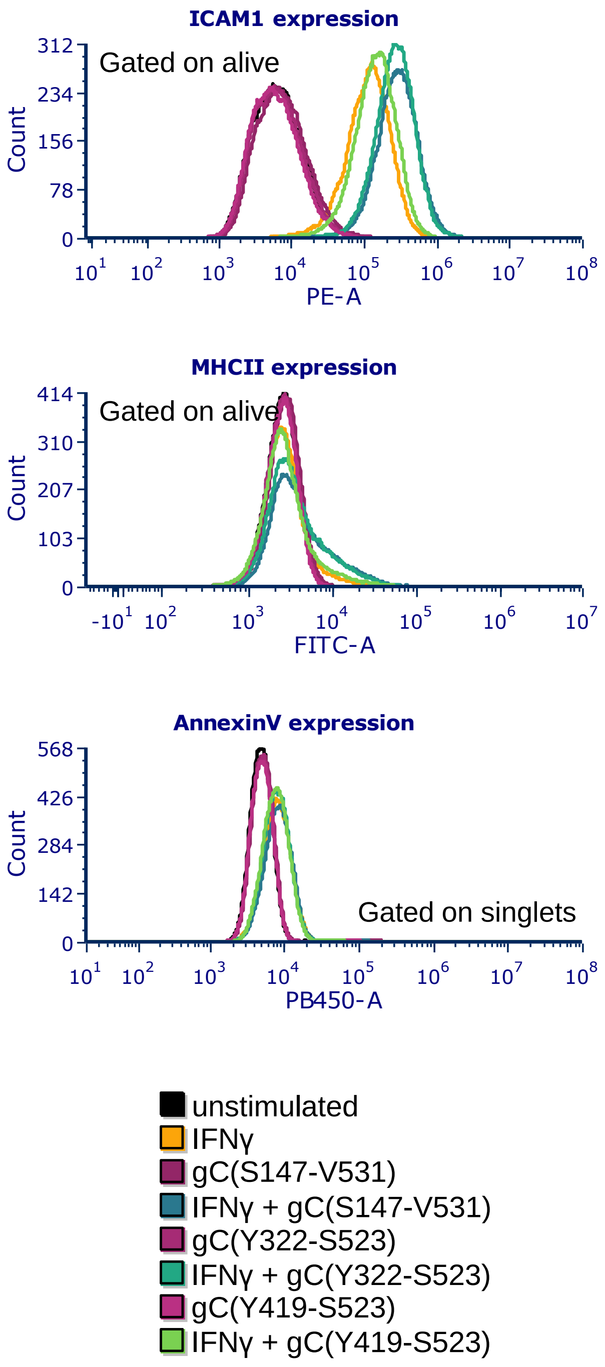

# Dot plots for Figure 3c

## Representative histograms

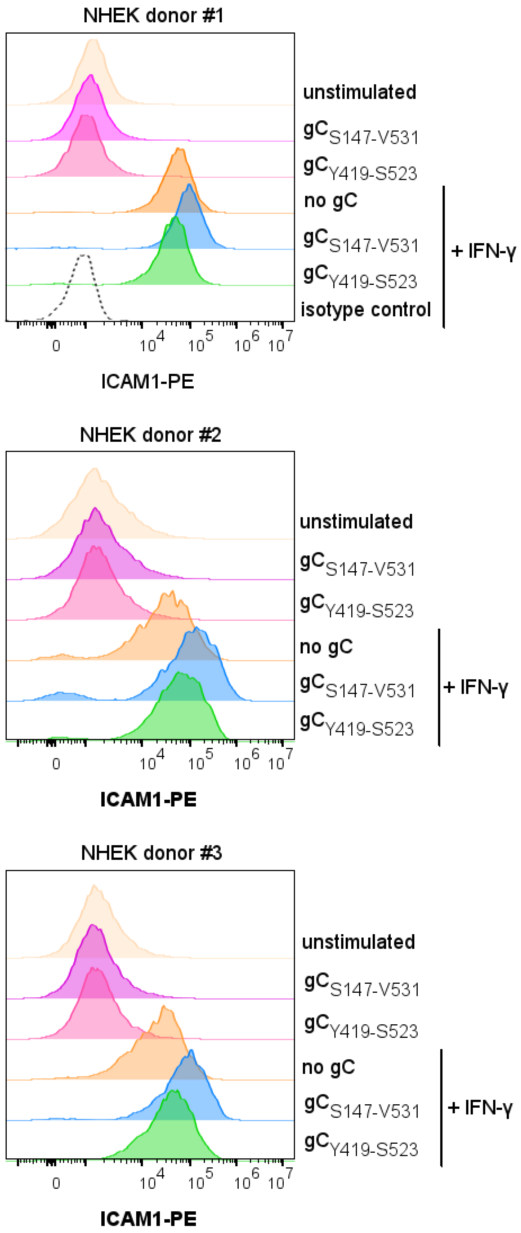

# Dot plots for Figure 3d,e

Representative histograms

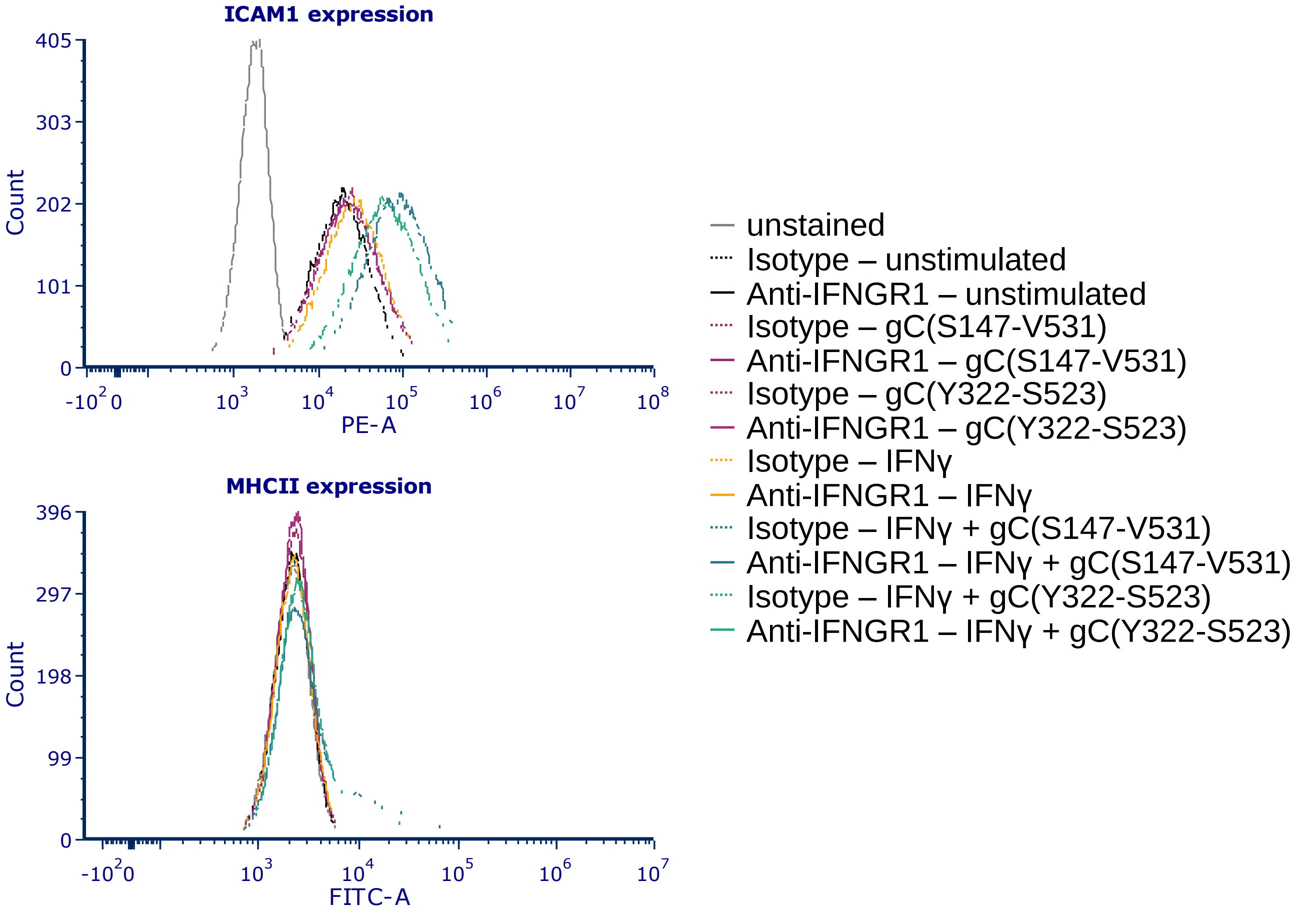

# Dot plots for Figure 7a-d

## Representative dot plots – HaCaT + Jurkat culture

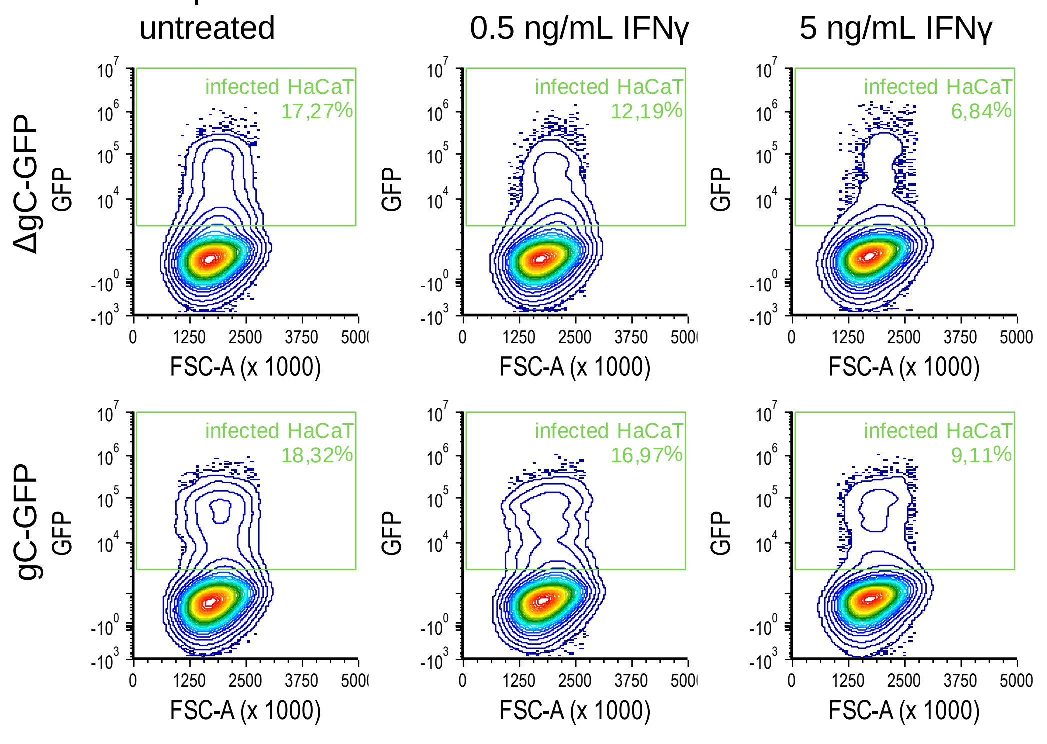


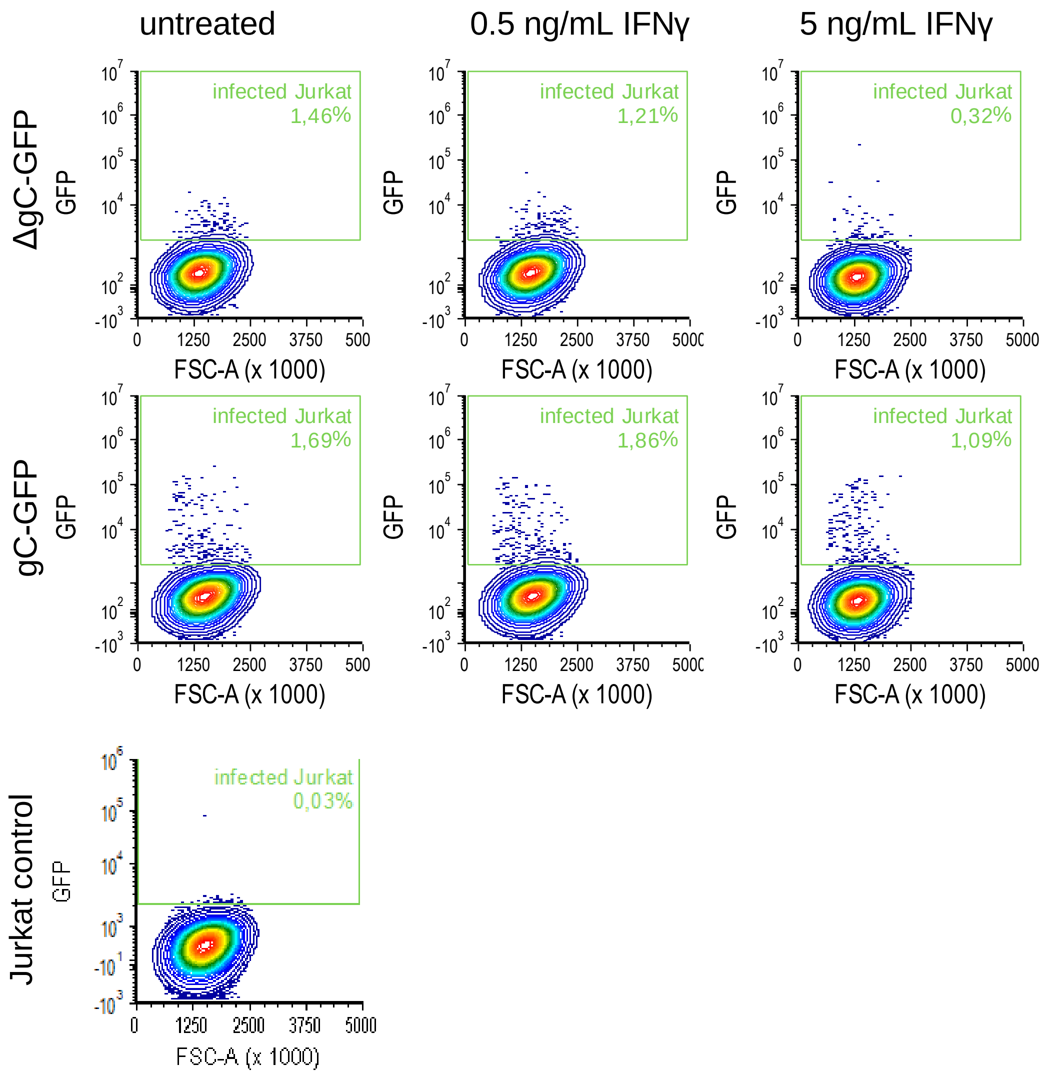

# Dot plots for Figure 7e-h

## Representative dot plots – HaCaT + PBMC culture

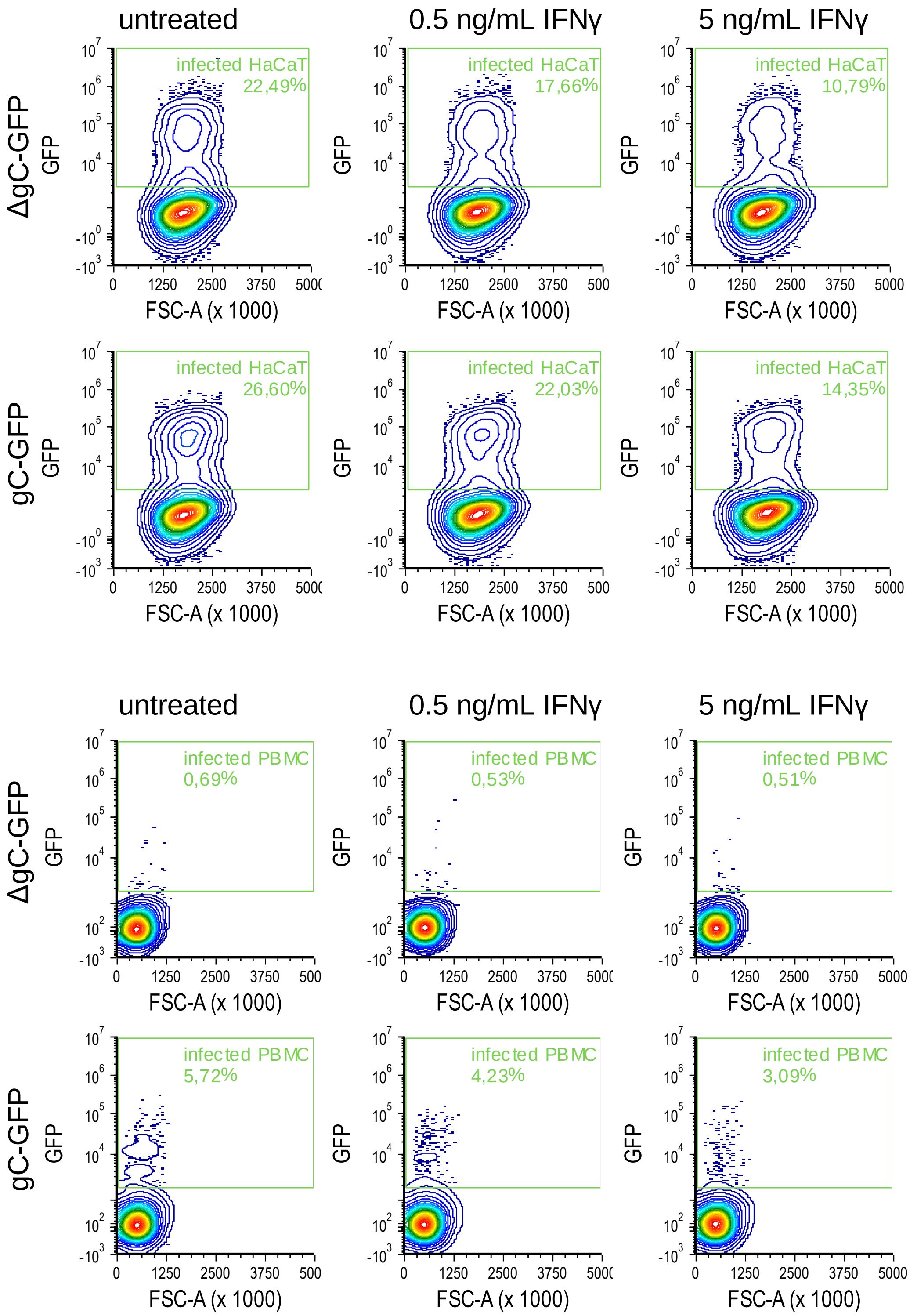

# Dot plots for Supplementary Figure 9

## Representative histograms

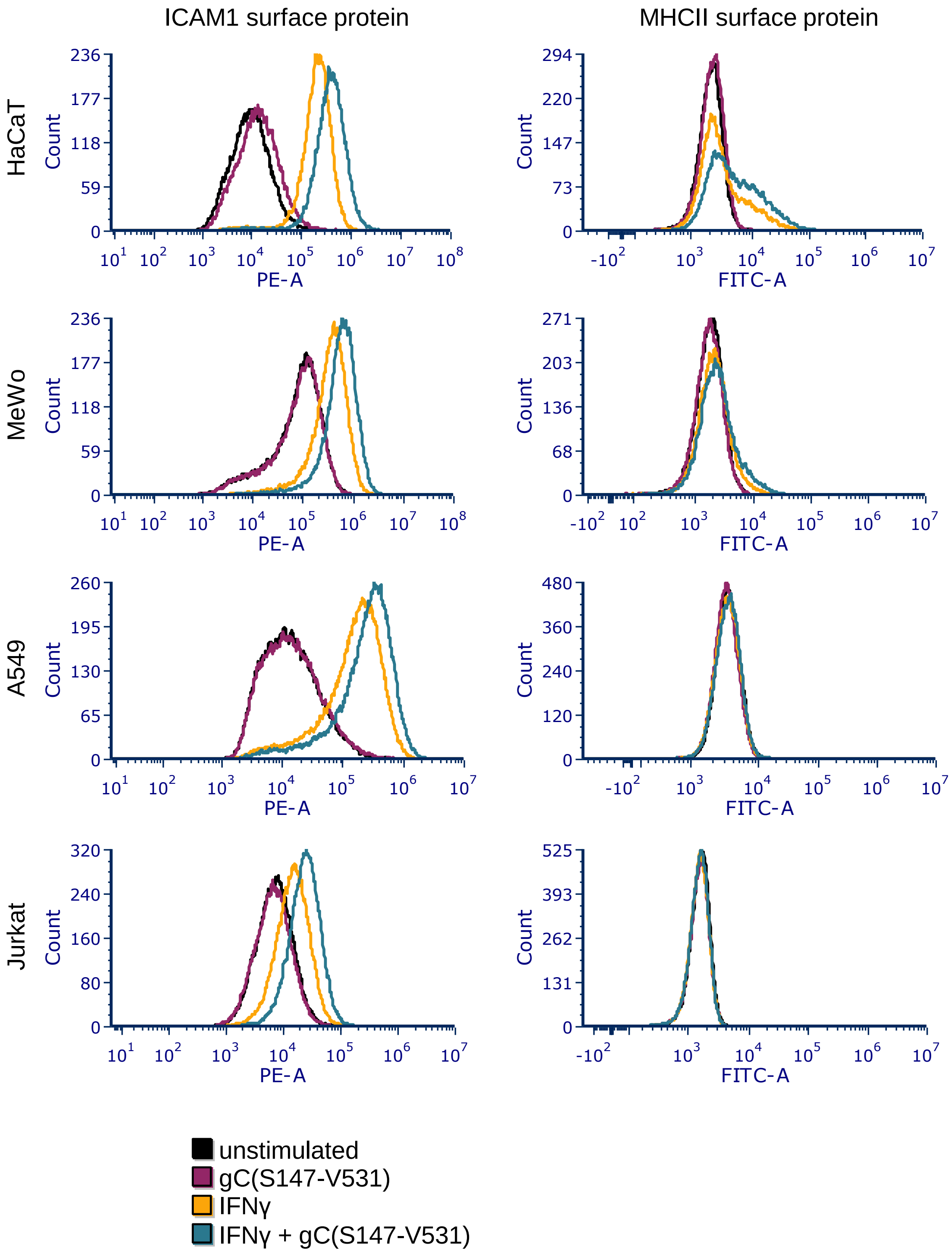

# Dot plots for Supplementary Figure 12a

## Representative histograms

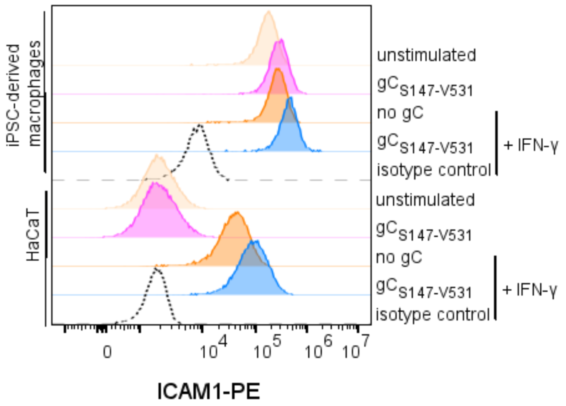


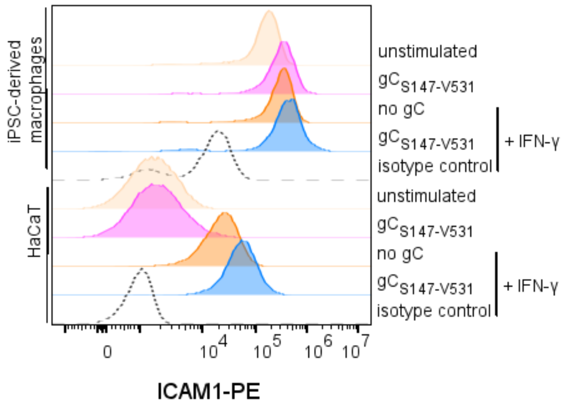

# Dot plots for Supplementary Figure 15

Representative dot plots and histogram

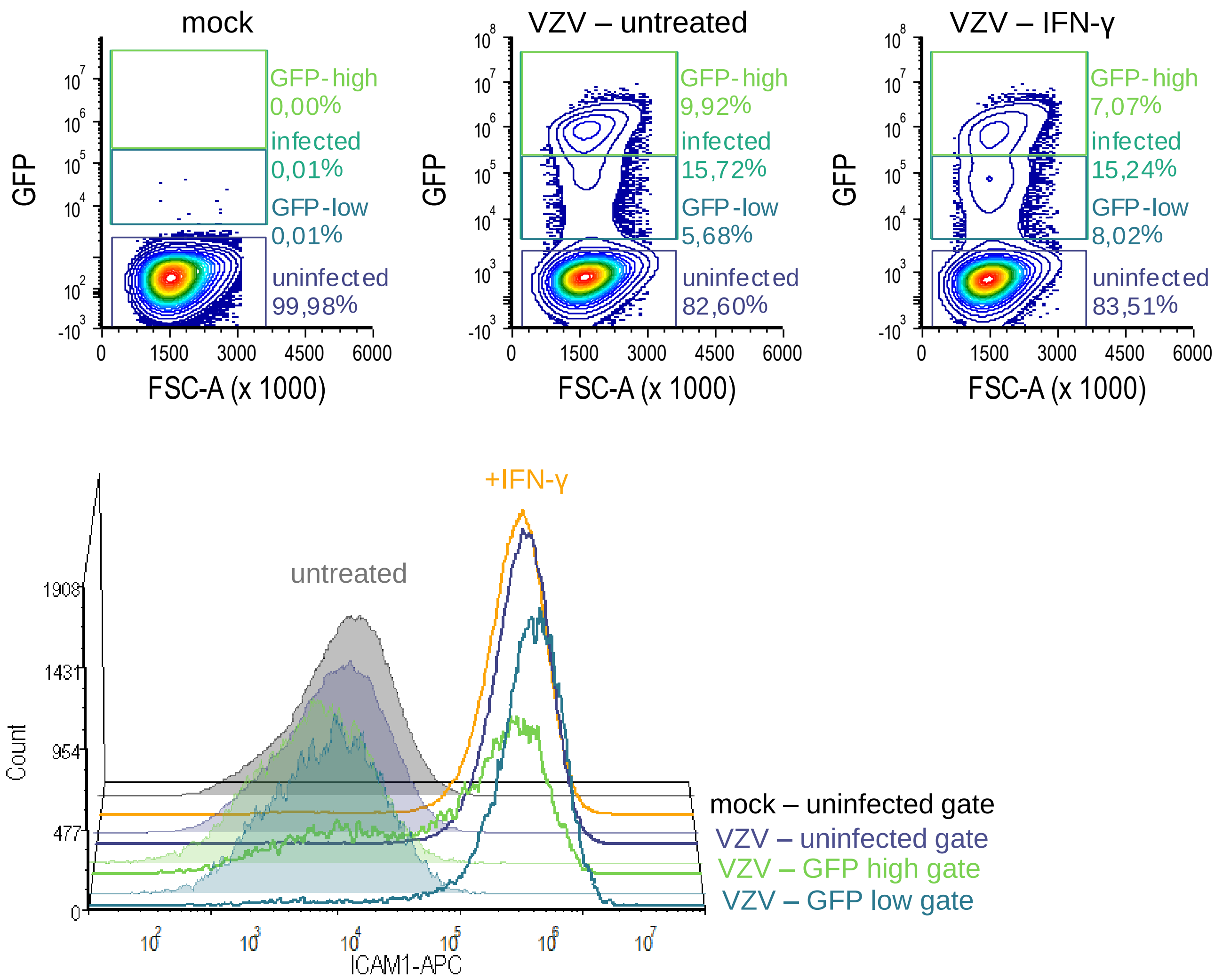

Supplement: Supplementary file 1 — Supplementary Information [file 41467_2024_49657_MOESM1_ESM.pdf]
