# Supplementary material for: Viral modulation of type II interferon increases T cell adhesion and virus spread
Source: Nat Commun. 2024 Jun 22;15:5318. doi: 10.1038/s41467-024-49657-4 (PMC11193720; doi:10.1038/s41467-024-49657-4)
Supplement: Supplementary file 3 — Reporting Summary [file 41467_2024_49657_MOESM3_ESM.pdf]

nature portfolio

Corresponding author(s): Abel Viejo-Borbolla

Last updated by author(s): May 22, 2024

Reporting Summary

Nature Portfolio wishes to improve the reproducibility of the work that we publish. This form provides structure for consistency and transparency in reporting. For further information on Nature Portfolio policies, see our Editorial Policies and the Editorial Policy Checklist.

Statistics

For all statistical analyses, confirm that the following items are present in the figure legend, table legend, main text, or Methods section.

n/a	Confirmed	
☐	☒	The exact sample size (*n*) for each experimental group/condition, given as a discrete number and unit of measurement
☐	☒	A statement on whether measurements were taken from distinct samples or whether the same sample was measured repeatedly
☐	☒	The statistical test(s) used AND whether they are one- or two-sided<br>*Only common tests should be described solely by name; describe more complex techniques in the Methods section.*
☒	☐	A description of all covariates tested
☐	☒	A description of any assumptions or corrections, such as tests of normality and adjustment for multiple comparisons
☐	☒	A full description of the statistical parameters including central tendency (e.g. means) or other basic estimates (e.g. regression coefficient) AND variation (e.g. standard deviation) or associated estimates of uncertainty (e.g. confidence intervals)
☐	☒	For null hypothesis testing, the test statistic (e.g. *F*, *t*, *r*) with confidence intervals, effect sizes, degrees of freedom and *P* value noted<br>*Give P values as exact values whenever suitable.*
☒	☐	For Bayesian analysis, information on the choice of priors and Markov chain Monte Carlo settings
☒	☐	For hierarchical and complex designs, identification of the appropriate level for tests and full reporting of outcomes
☒	☐	Estimates of effect sizes (e.g. Cohen's *d*, Pearson's *r*), indicating how they were calculated

Our web collection on statistics for biologists contains articles on many of the points above.

Software and code

Policy information about availability of computer code

Data collection	Biacore X100 system (GE Healthcare, Cytiva), Biacore S200 system (Cytiva), WAVEdelta system (Creoptix), ÄKTApure 25 system (Cytiva), Cytoflex S (Beckman Coulter), ChemiDoc MP Imaging system (Bio-Rad), Cytation3 plate reader (BioTek), Gel iX20 Imager (INTAS Science Imaging), Zeiss LSM 980 with Airyscan 2 (Zeiss), qPCR Tower3 from Analytic Jena AG, Illumina MiSeq, Oxford Nanopore MinIon Mk1B
Data analysis	Biacore X100 Control software and Evaluation software 2.0.1 (GE Healthcare), Cell Profiler 4.0.7 (Broad Institute, Inc), Cyt Expert 2.4 Beckman Coulter, FCS Express 6 and 7 (DeNovo Software), Gen5 Image+ 2.09 (BioTek), GraphPad Prism 9 (GraphPad Software), Image Lab 6.0.1, (Bio-Rad), Qlucore Omics Explorer 3.8 (Qlucore), qPCRsoft 4.1.3.0 (Analytik Jena AG), RStudio 2021.09.2 (RStudio, PBC), WAVEcontrol 4.3.4 (Creoptix), FlowJo_v10.10.0 (BD, Becton, Dickinson & Company), Image J 1.53a (National Institutes of Health), MiSeq Control Software 2.6.2.1, MinKNOW V 23.11.5

For manuscripts utilizing custom algorithms or software that are central to the research but not yet described in published literature, software must be made available to editors and reviewers. We strongly encourage code deposition in a community repository (e.g. GitHub). See the Nature Portfolio guidelines for submitting code & software for further information.

Data

Policy information about availability of data

All manuscripts must include a data availability statement. This statement should provide the following information, where applicable:

- Accession codes, unique identifiers, or web links for publicly available datasets
- A description of any restrictions on data availability
- For clinical datasets or third party data, please ensure that the statement adheres to our policy

The RNAseq datasets generated and analysed in the current study are available in the European Nucleotide Archive repository, with the following accession number PRJEB61951 (https://www.ebi.ac.uk/ena/browser/view/PRJEB61951). The genome sequences of the viruses generated in this report are available at GenBank with the following accession numbers: pOka-Δ57-GFP, PP378487 (https://www.ncbi.nlm.nih.gov/nuccore/PP378487); pOka-ΔgC-GFP, PP378488 (https://www.ncbi.nlm.nih.gov/nuccore/PP378488); pOka-gC-GFP, PP378489 (https://www.ncbi.nlm.nih.gov/nuccore/PP378489). iPSC lines used in this study can be obtained after signing appropriate material transfer agreements. RNA fold changes comparing the different groups and their significances were calculated using DESeq2 can be found in Source Data 1. All data presented in graphs within figures is included in Source Data 2. The uncropped western blots for Figure 4a can be found in Source Data 3 and the uncropped gels and western blots for Supplementary Figures 1 and 8 can be found in Supplementary Information (Supplementary Figures 17 and 18). The gating strategy and the dot plots are presented as Supplementary Information file 1 and 2, respectively. Source Data are provided with this paper.

Research involving human participants, their data, or biological material

Policy information about studies with human participants or human data. See also policy information about sex, gender (identity/presentation), and sexual orientation and race, ethnicity and racism.

Reporting on sex and gender	We employed peripheral blood mononuclear cells (PBMCs) provided by the Institute of Transfusion Medicine, Hannover Medical School, Hannover, Germany that obtained them from healthy donors in an anonymized way. Therefore, we do not have access to information regarding sex and gender.<br>We employed foreskin normal human epithelial keratinocytes (NHEK) obtained from surgical residuals from children. All donors were therefore male.
Reporting on race, ethnicity, or other socially relevant groupings	N/A, we do not have access to information regarding race, ethnicity or other socially relevant grouping of the blood donors. The NHEK were obtained from surgical foreskin residuals from anonymised children. Therefore, we do not have this information
Population characteristics	N/A, we do not have access to such information regarding PBMCs. All NHEK donors were children
Recruitment	No recruitment was performed for this study. The PBMCs from anonymised healthy blood donors were provided by the Institute of Transfusion Medicine, MHH, Germany. Similarly, there was no recruitment to obtain NHEK
Ethics oversight	PBMCs: Ethics committee of HAnnover Medical school #2519-2014 and #10476_BO_K_2022.<br>NHEK were obtained from surgical residuals from anonymised individuals and therefore there was no need for an ethical approval

Note that full information on the approval of the study protocol must also be provided in the manuscript.

Field-specific reporting

Please select the one below that is the best fit for your research. If you are not sure, read the appropriate sections before making your selection.

☒ Life sciences ☐ Behavioural & social sciences ☐ Ecological, evolutionary & environmental sciences

For a reference copy of the document with all sections, see nature.com/documents/nr-reporting-summary-flat.pdf

Life sciences study design

All studies must disclose on these points even when the disclosure is negative.

Sample size	The sample size was determined based on previous experiments performed in the lab
Data exclusions	There was no exclusion of data with the exception of one data point in Supp. Fig 8b, 6 hours post-incubation with IFNg + gC since there was very little protein following protein precipitation with TCA. This did not affect the conclusions of that figure. This is indicated in Source data 2
Replication	If not stated otherwise, all experiments were performed at least three times (biological replicates) with similar results
Randomization	There was no predetermined allocation into experimental groups
Blinding	Blinding was not relevant in this study because of the observational measurements by software algorithms

Reporting for specific materials, systems and methods

We require information from authors about some types of materials, experimental systems and methods used in many studies. Here, indicate whether each material, system or method listed is relevant to your study. If you are not sure if a list item applies to your research, read the appropriate section before selecting a response.

Materials & experimental systems

n/a	Involved in the study
☐	☒ Antibodies
☐	☒ Eukaryotic cell lines
☒	☐ Palaeontology and archaeology
☒	☐ Animals and other organisms
☒	☐ Clinical data
☒	☐ Dual use research of concern
☒	☐ Plants

Methods

n/a	Involved in the study
☒	☐ ChIP-seq
☐	☒ Flow cytometry
☒	☐ MRI-based neuroimaging

Antibodies

Antibodies used

BD Pharmingen™ Purified NA/LE Mouse IgG1 κ Isotype Control, Clone 107.3, BD Bioscience #554721. The 107.3 clone has an unknown specificity. Trinitrophenal (TNP), the immunogen, is a hapten not expressed on human or mouse cells. In the absence of specific binding, this antibody may bind non-specifically to Fc receptors. The immunoglobulin from clone 107.3 was selected as an isotype control following screening for low background on a variety of mouse and human tissues. This material is suitable as an isotype control for matching cytokine-neutralizing antibodies used in bioassay or as a negative control for in vitro and in vivo functional studies.
BD Pharmingen™ Purified NA/LE Mouse Anti-Human CD119, Clone GIR-208, BD Bioscience #557531. Binding of 125I-labeled GIR-208 antibody to IFN-γRα+ cells is reported to be specifically inhibited in the presence of excess IFN-γ. GIR-208 does not cross react with IFN-γ as tested by ELISA. The ability of this antibody to bind to IFN-γ receptors of species other than human has not been determined. The immunogen used to generate this hybridoma was human IFN-γRα purified from human placenta. The GIR- 208 has been reported to block the binding of 125I-human IFN-γ to IFN-γRα+ cells as well as purified, soluble human IFN-γRα. GIR-208 is a neutralizing antibody that has been shown to neutralize the anti-viral activity of IFN-γ on WISH cells in a dose-dependent fashion.
Anti-beta Actin Monoclonal Antibody (15G5A11/E2), Thermo Scientific #MA 1-140. This Antibody was verified by Cell treatment to ensure that the antibody binds to the antigen stated.
Anti-CD54/ICAM-1 Antibody, Cell Signaling Technology #4915S. CD54/ICAM-1 Antibody detects endogenous levels of total CD54 (ICAM-1) protein. The antibody does not cross-react with other IgSF adhesion molecules. Species Reactivity: Human.
IRDye® 800CW Goat anti-Rabbit IgG Secondary Antibody, LI-COR #925-32211. Isolation of specific antibodies was accomplished by affinity chromatography using pooled rabbit IgG covalently linked to agarose. Based on ELISA and flow cytometry, this antibody reacts with the heavy and light chains of rabbit IgG, and with the light chains of rabbit IgM and IgA. This antibody was tested by dot blot and and/or solid-phase adsorbed for minimal cross-reactivity with human, mouse, rat, sheep, and chicken serum proteins, but may cross-react with immunoglobulins from other species. The conjugate has been specifically tested and qualified for Western blot and In-Cell Western™ Assay applications.
IRDye® 680RD Goat anti-Mouse IgG Secondary Antibody, LI-COR #925-68070. Isolation of specific antibodies was accomplished by affinity chromatography using pooled mouse IgG covalently linked to agarose. Based on ELISA and flow cytometry, this antibody reacts with the heavy and light chains of mouse IgG1, IgG2a, IgG2b, and IgG3, and with the light chains of mouse IgM and IgA. This antibody was tested by dot blot and and/or solid-phase adsorbed for minimal cross-reactivity with human, rabbit, goat, rat, and horse serum proteins, but may cross-react with immunoglobulins from other species. The conjugate has been specifically tested and qualified for Western blot and In-Cell Western™ Assay applications.
PE anti-human CD54 Antibody (clone HA58), Biolegend #353106. Verified Reactivity, Human. Each lot of this antibody is quality control tested by immunofluorescent staining with flow cytometric analysis. Clone HA58 recognizes an epitope located in the extracellular D1 domain of CD54.
APC anti-human CD54 Antibody (clone HA58), Biolegend #353112. Verified Reactivity, Human. Each lot of this antibody is quality control tested by immunofluorescent staining with flow cytometric analysis.
panHLA-ClassII (HKB1), ImmunoTools #21629233X2. The antibody recognises all HLA-Class II antigens, the 3 major (HLA-DP, -DQ and -DR) and 2 minor Major Histocompatibility Complex MHC class II proteins (HLA-DM and -DO). The genes of the class II combine to form heterodimeric (αβ) protein receptors that are typically expressed on the surface of antigen presenting cells (APCs): dendritic cells B-cells and macrophages. HLA class II is presented on activated T-cells. HLA class II molecules present exogenously derived antigen to the T cell receptor (TCR) on CD4 + T lymphocytes.
Anti-Phospho-Stat1 (Tyr701) Rabbit mAb, Cell Signaling Technology #9167S. The antibody detects endogenous levels of Stat1 only when phosphorylated at tyrosine 701. The antibody detects phosphorylated tyrosine 701 of p91 Stat1 and also the p84 splice variant. It does not cross-react with the corresponding phospho-tyrosines of other Stat proteins. Species reactivity: Human, Mouse.
Polyclonal Goat Anti-Rabbit Immunoglobulins/HRP, Dako #P0448. Affinity-isolated goat antibody conjugated with horseradish peroxidase (HRP) of very high specific enzymatic activity. Cross-reactions as determined by ELISA for the unconjugated antibody: Human, mouse and rat immunoglobulins, less than 1%.
Donkey anti-Rabbit IgG (H+L) Highly Cross-Adsorbed Secondary Antibody, Alexa Fluor™ 555, Invitrogen #A-31572. These donkey anti-rabbit IgG (H+L)whole secondary antibodies have been affinity-purified and show minimum cross-reactivity.
APC anti-human CD11a (LFA-1α chain) Antibody (clone HI111), BioLegend #301212, Verified reactivity. Human. Each lot of this antibody is quality control tested by immunofluorescent staining with flow cytometric analysis. HI111 is specific for the closed confirmation of the integrin.
APC Mouse IgG1, κ Isotype Ctrl Antibody (clone MPOC-21), BioLegend #400119. The MOPC-21 immunoglobulin has unknown

specificity. The isotype of this antibody is mouse IgG1, κ. This antibody was chosen as an isotype control after screening on a variety of resting, activated, live, and fixed mouse, rat and human tissues.
Mouse IgG1 kappa Isotype Control (P3.6.2.8.1), PE, eBioscience™ (Invitrogen) #12-4714-82.
FITC Mouse IgM, κ Isotype Ctrl Antibody (clone MM-30), BioLegend #401606. The isotype of MM-30 immunoglobulin is mouse IgM, κ. This antibody was chosen as an isotype control after screening on a variety of resting, activated, live, and fixed mouse, rat and human tissues.
Mouse monoclonal anti-ORF14, clone VZ 14.12 (CapRi, Croatia)specifically binds to VZV glycoprotein C (gC) the product of VZV ORF14. We used this antibody to detect gC in infected cells.

Validation

Antibodies were obtained from commercial sources that carry validation process, as indicated above. We also validated them using appropriate positive and negative controls

Eukaryotic cell lines

Policy information about cell lines and Sex and Gender in Research

Cell line source(s)

A549 is an epithelial cell line isolated from lung tissue of a caucasian male with lung cancer and was provided by Thomas Pietschmann (Twincore, Hannover, Germany).
HaCaT cells are in vitro spontaneously transformed keratinocytes derived from histologically normal skin from a caucasian male. These cells were provided by Beate Sodeik (Institute of Virology, MHH, Germany).
Jurkat E6.1 originate from a male patient with acute T cell leukemia and were kindly provided by Martin Messerle (Institute of Virology, MHH, Germany).
Jurkat LFA-1 KO cells were a gift from Carsten Münk (Düsseldorf University Hospital, Germany) and have been described in Hain et al. 2018 Sci Rep.
MeWo cells were derived from a male patient suffering malignant melanoma and were purchased from ATCC (HTB-65TM).
Peripheral blood mononuclear cells (PBMCs) from anonymized healthy blood donors were obtained from the Institute of Transfusion Medicine, MHH, Germany. The sex of the donors was unknown.
Schneider's Drosophila melanogaster Line 2 (S2) cells (ATCC No: CRL-1963) were derived from embryonic tissue of the fruit fly Drosophila melanogaster and were purchased from Thermo Scientific.
We used male and female iPSC lines (MHHi015-A is female, LiPSC-GR1.1 is male), and that the lines were either quantified by STR analysis or the use of iPSC-derived cells which have been compared to diseased donor cells
NHEK were obtained from foreskin surgical residuals from anonymous children. The purity of keratinocytes was verified by the expression of the epithelial marker cytokeratin (mouse anti-human cytokeratin antibody, clone: MNF-116, DakoCytomation, Hamburg, Germany) and the fibroblast-specific marker ASO2 (CD90, Dianova GmbH, Hamburg, Germany). All cells (more than 95%) were found to be uniformly positive for cytokeratin but not for CD90.
ARPE-19 are human retinal pigment epithelial cell line (ATCC-CRL-2302). The cells were provided by Martin Messerle (Institute of Virology, Hannover Medical School, Germany).

Authentication

None of the cells were autenthicated with exception of the iPSC lines that were subjected to STR analysis and NHEK (see below)
NHEK: The purity of keratinocytes was verified by the expression of the epithelial marker cytokeratin (mouse anti-human cytokeratin antibody, clone: MNF-116, DakoCytomation, Hamburg, Germany) and the fibroblast-specific marker ASO2 (CD90, Dianova GmbH, Hamburg, Germany). All cells (more than 95%) were found to be uniformly positive for cytokeratin but not for CD90.

Mycoplasma contamination

All cell lines were regularly tested for mycoplasma and were negative

Commonly misidentified lines
(See ICLAC register)

No commonly misidentified lines were used.

Plants

Seed stocks

Report on the source of all seed stocks or other plant material used. If applicable, state the seed stock centre and catalogue number. If plant specimens were collected from the field, describe the collection location, date and sampling procedures.

Novel plant genotypes

Describe the methods by which all novel plant genotypes were produced. This includes those generated by transgenic approaches, gene editing, chemical/radiation-based mutagenesis and hybridization. For transgenic lines, describe the transformation method, the number of independent lines analyzed and the generation upon which experiments were performed. For gene-edited lines, describe the editor used, the endogenous sequence targeted for editing, the targeting guide RNA sequence (if applicable) and how the editor was applied.

Authentication

Describe any authentication procedures for each seed stock used or novel genotype generated. Describe any experiments used to assess the effect of a mutation and, where applicable, how potential secondary effects (e.g. second site T-DNA insertions, mosiacism, off-target gene editing) were examined.

Flow Cytometry

Plots

Confirm that:

☒ The axis labels state the marker and fluorochrome used (e.g. CD4-FITC).

☒ The axis scales are clearly visible. Include numbers along axes only for bottom left plot of group (a 'group' is an analysis of identical markers).

☒ All plots are contour plots with outliers or pseudocolor plots.

☐ A numerical value for number of cells or percentage (with statistics) is provided.

Methodology

Sample preparation	We have different sample preparation procedures that are explained in the corresponding Methods section
Instrument	Cytoflex S (Beckmann Coulter)
Software	Cyt Expert 2.4 Beckman Coulter, FCS Express 6 (DeNovo Software), FlowJo_v10.10.0 (BD, Becton, Dickinson & Company)
Cell population abundance	Cell lines showed a uniform population. PBMCs were not further discriminated into separate populations<br>NHEK were characterized as keratinocytes by the expression of epithelial marker cytokeratin (mouse anti-human cytokeratin antibody, clone: MNF-116, DakoCytomation, Hamburg, Germany) and the fibroblast-specific marker ASO2 (CD90, Dianova GmbH, Hamburg, Germany). All cells (more than 95%) were found to be uniformly positive for cytokeratin but not for CD90
Gating strategy	Gating for ICAM1, MHCII and LFA-1:<br>FSC-H/SSC-H or alternatively FSC-A/SSC-A to gate on main cell population --> FSC-H/FSC-W or alternatively SSC-A/SSC-H to gate on single cells --> FSC-H/A/APC-A750-A to gate on alive cells--> histogram for PE, APC, FITC; for infected (GFP-expressing) cells after gating on single, alive cells --> FSC-A/FITC-A clear formation of 2 populations GFP high, GFP low (GFP positive ones = infected) and GFP negative (= uninfected)<br>Gating VZV spread:<br>FSC-A/SSC-A to gate for HaCaT population --> FSC-A/PB450-A to gate for Hoechst negative cells --> FSC-H/FSC-W to gate for single cells --> FSC-A/FITC-A to identify GFP positive HaCaT cells<br>FSC-A/SSC-A to gate for Jurkat population --> FSC-A/PB450-A to gate for Hoechst positive cells --> FSC-H/FSC-W to gate for single cells --> FSC-A/FITC-A to identify GFP positive Jurkat cells<br>FSC-A/SSC-A to gate for PBMC population --> FSC-A/PB450-A to gate for Hoechst positive cells --> FSC-A/FITC-A to identify GFP positive PBMC cells

☒ Tick this box to confirm that a figure exemplifying the gating strategy is provided in the Supplementary Information.
