# Supplementary material for: Viral modulation of type II interferon increases T cell adhesion and virus spread
Source: Nat Commun. 2024 Jun 22;15:5318. doi: 10.1038/s41467-024-49657-4 (PMC11193720; doi:10.1038/s41467-024-49657-4)
Supplement: Supplementary file 4 — Source Data [file 41467_2024_49657_MOESM4_ESM.zip › R3_Source_Data_3.pdf]

Source Data 3: Uncropped blots for Figure 4a

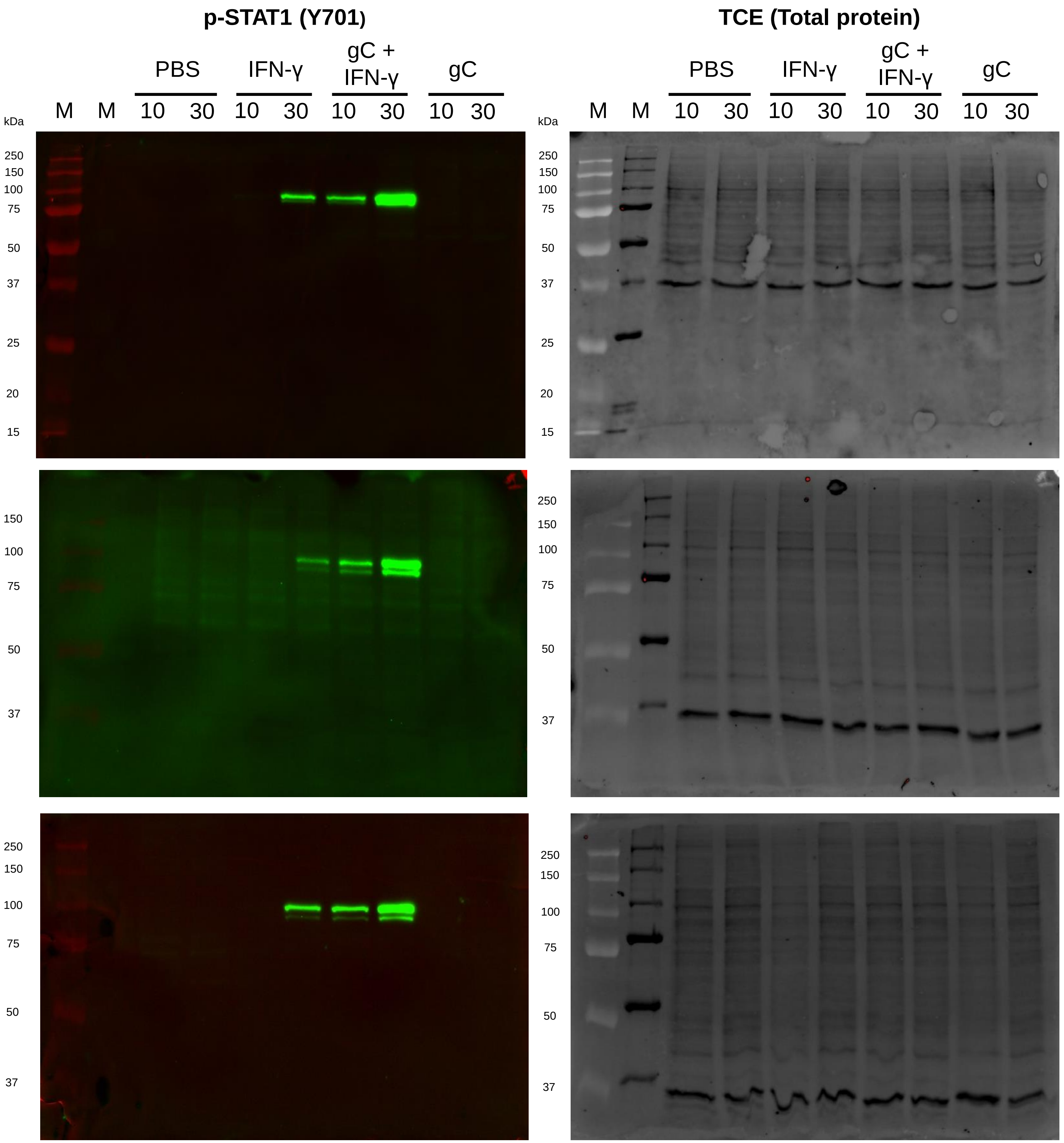

Blots showing p-STAT1 (left) and total protein (TCE staining) from three biological experiments
